# Comprehensive Analysis to Identify MAGEA3 Expression Correlated With Immune Infiltrates and Lymph Node Metastasis in Gastric Cancer

**DOI:** 10.3389/fonc.2021.784925

**Published:** 2021-12-14

**Authors:** Jinji Jin, Jianxin Tu, Jiahuan Ren, Yiqi Cai, Wenjing Chen, Lifang Zhang, Qiyu Zhang, Guanbao Zhu

**Affiliations:** ^1^ Department of Gastrointestinal Surgery, The First Affiliated Hospital of Wenzhou Medical University, Wenzhou, China; ^2^ Department of Rheumatology, The First Affiliated Hospital of Wenzhou Medical University, Wenzhou, China; ^3^ Department of Medical Microbiology and Immunology, Wenzhou Medical University, Wenzhou, China; ^4^ Department of Hepato-Bilio-Pancreatic Surgery, The First Affiliated Hospital of Wenzhou Medical University, Wenzhou, China

**Keywords:** gastric cancer, MAGEA3, immune infiltrates, lymph node metastasis (LNM), immunotherapy

## Abstract

Gastric cancer (GC) is an aggressive malignant tumor and causes a significant number of deaths every year. With the coming of the age of cancer immunotherapy, search for a new target in gastric cancer may benefit more advanced patients. Melanoma-associated antigen-A3 (MAGEA3), one of the members of the cancer-testis antigen (CTA) family, was considered an important part of cancer immunotherapy. We evaluate the potential role of MAGEA3 in GC through the TCGA database. The result revealed that MAGEA3 is upregulated in GC and linked to poor OS and lymph node metastasis. MAGEA3 was also correlated with immune checkpoints, TMB, and affected the tumor immune microenvironment and the prognosis of GC through CIBERSORT, TIMER, and Kaplan-Meier plotter database analysis. In addition, GSEA-identified MAGEA3 is involved in the immune regulation of GC. Moreover, the protein-protein interaction (PPI) networks of MAGEA3 were constructed through STRING database and MAGEA3-correlated miRNAs were screened based on the joint analysis of multiple databases. In terms of experimental verification, we constructed pET21a (+)/MAGEA3 restructuring plasmids and transformed to *Escherichia coli* Rosetta. MAGEA3 protein was used as an antigen after being expressed and purified and can effectively detect the specific IgG in 93 GC patients’ serum specimens with 44.08% sensitivity and 92.54% specificity. Through further analysis, the positive rate of MAGEA3 was related to the stage and transfer number of lymph nodes. These results indicated that MAGEA3 is a novel biomarker and correlated with lymph node metastasis and immune infiltrates in GC, which could be a new target for immunotherapy.

## Introduction

Gastric cancer (GC) is an aggressive and devastating disease, with more than 1 million new cases a year, and remains the fourth cause of cancer-related death, although the mortality and mortality were declining gradually (1). Despite the progress made in the management of gastric cancer over decades, prognosis remains poor, and the 5-year survival in patients with metastatic disease is 5.3% (2). Many factors contribute to the risk of gastric cancer; infection with *Helicobacter pylori* is the main cause and confirmed as the first biological carcinogen by WHO (3–5). Epstein-Barr virus (EBV) infection (6), environmental and genetic factors, obesity, and smoking also contribute to the development of stomach cancer (7, 8). At present, carcinoembryonic antigens including CEA and CA19-9 are the most widely used gastric cancer detection markers in clinical practice (9, 10). However, these markers lack the sensitivity and specificity needed to assess the diagnosis and prognosis of gastric cancer; thus, many other tumor markers have been discovered and proved their potential efficacy as diagnostic and prognostic tools in gastric cancer. However, these markers are also having problems, such as, insufficient sensitivity that needs further clinical verification (11). Traditional cancer therapies like surgery and chemoradiation therapy are limited to the treatment of advanced gastric cancer patients, so innovative approaches are desperately needed. Immunotherapy offers a different approach and is an alternative treatment option for those patients, and many clinical trials are in progress (12). The purpose of this study is to find a target that plays a role in detection and immunotherapy.

Cancer testis antigens (CTA) are antigens that are usually only expressed in testis and placenta and various tumor types (13). Melanoma-associated antigen-A3 (MAGEA3), as a main member of CTA, is located on chromosome Xq28. The expression of MAGEA3 is modulated by DNA methylation or histone acetylation (14–16). Many research have reported the abnormal expression of MAGEA3 in many tumor types (17–21). The characteristics of differential expression in normal and cancer tissues make MAGEA3 an ideal target for antitumor vaccines and carried out various clinical trials (22–25). However, the two largest phase III clinical trials targeting MAGEA3 immunotherapeutic as an adjuvant therapy for stage III melanoma and nonsmall cell lung cancer failed (26, 27), which is stagnating the progress of immunotherapeutic, and research on MAGEA3 also have declined. In our previous study, we have identified epitopes from MAGEA3 protein and found that patients with gastric cancer had higher reactivity to these epitopes (28); we also found that MAGEA3 multiepitope vaccine can induce humoral and cellular immune responses in mice (29), so we still believe MAGEA3 is an important target for GC diagnosis and immunotherapy. In this research, we analyzed the relationship between MAGEA3 and gastric cancer patients’ prognosis through the Cancer Genome Atlas (TCGA) database and investigated the effect of MAGEA3 expression on immune cell infiltration, further screening out MAGEA3-related proteins and interacting miRNA. We further use purified MAGEA3 protein for the detection of specific antibodies in the serum of GC patients to prove that MAGEA3 is related to the progression of gastric cancer. Our findings provide novel insights into the role of MAGEA3 in GC, thereby highlighting the underlying mechanism of MAGEA3 influencing immune cell interaction with tumors and providing preliminary preparations for the detection and immunotherapy of MAGEA3 in gastric cancer.

## Methods

### Gastric Cancer Patients in TCGA

RNA sequence profiles and clinical data of 375 GC patients and 32 normal controls were downloaded through the TCGA database (https://genome-cancer.ucsc.edu/). Subsequently, analysis includes clean data and cancer dataset divided into 2 groups by median.

### TIMER Analysis

TIMER is a comprehensive website (https://cistrome.shinyapps.io/timer/) that can analyze the differences in gene expression and the levels of immune invasion in different tumors (30). We first explored the expression of MAGEA3 in pan-cancer. “SCNA module,” “Gene module,” and “Survival module” were then applied to evaluate the association between MAGEA3 and immune infiltration and clinical outcome. Finally, the correlation of MAGEA3 with the markers of immune cells in GC was verified.

### Kaplan-Meier Plotter Analysis

The Kaplan-Meier plotter website (http://kmplot.com/) can explore the impact of gene on patient survival in more than 20 cancer types, including gastric cancer (*n* = 1,440). We explore the association between MAGEA3 expression and prognosis of GC in the related immune cell subgroups.

### Immune Checkpoints and TMB and MSI Analyses

To predict the part of patients who would benefit from immune checkpoint inhibitors (ICI) or agonists, we compared the differential expression of immune checkpoints between MAGEA3 high group and MAGEA3 low group. PDCD1 (PD-1), CD274 (PD-L1), CTLA4, SIGLEC15, IDO1, HAVCR2, LAG3, and PDCD1LG2 were chosen as immune checkpoints.

Tumor mutational burden (TMB) and microsatellite instability (MSI) have been viewed as biomarkers for predicting the therapeutic response to immunotherapy (31). We used Spearman’s correlation analysis to describe the correlation between nonnormal distributed quantitative variables.

### CIBERSORT Analysis

We used the CIBERSORT to analyze the normalized data filtered by Perl programming language and obtained the immune cell infiltration matrix. We then used “corrplot” package to draw a correlation heatmap to visualize the correlation of 22 types of infiltration and used “ggplot2” package to draw violin diagrams to visualize the differences in immune cell infiltration in different MAGEA3 groups.

### Gene Set Enrichment Analysis

Gene set enrichment analysis (GSEA) was performed using the Java GSEA desktop application. In this study, GSEA v4.1.0 software was used to identify immunological features in different MAGEA3 groups. The random combination was set for 1,000 times. NOM *p*-value <0.05 and FDR <0.05, |NES|>1 were considered significant enrichment.

### PPI Network Construction

The R package “Limma” was used to identify DEGs between MAGEA3 high group and MAGEA3 low group. An adjusted *p* < 0.05 and |log_2_FC|>1was used as cutoff values. The STRING website (http://string-db.org) (32) was used to construct an interactive network of DEGs and subsequently was visualized by Cytoscape software. We then filtered out the module that MAGEA3 was involved in through the MCODE plugin. We also listed MAGEA3-binding proteins with the experimental evidence identification based on the STRING database. Then screened out the possible proteins that may interact with MAGEA3 base on the intersection of the results of STRING and MCODE.

### Candidate miRNA Prediction

To predict the miRNAs that may target MAGEA3, five target gene prediction websites were analyzed, including, ENCORI, TargetScan, miRmap, mirDIP, and mircoT. We then used Venn to conduct an intersection analysis. The ENCORI website was use to further verify the miRNA in gastric cancer.

### Construction of pET21a(+)/MAGEA3 Recombinant Plasmid and Expression and Purification

The full-length gene sequence of MAGEA3 was optimized by a prokaryotic codon (www.jcat.de) and synthesized by Shanghai Biological Engineering Co., Ltd. MAGEA3 was then cloned into prokaryotic expression vector pET21a(+) to obtain a recombinant plasmid: pET21(+)/MAGEA3. In order to express recombinant proteins, *Escherichia coli* Rosetta transformed with positive plasmid was induced under 1 mmol/l IPTG at 30°C. Bacteria were then collected and washed once with PBS. The lysate was sonicated, and then centrifuged at 10,000×*g* for 10 min, and the supernatant was collected. The purification of MAGEA3 proteins were performed according to the procedure recommended by Qiagen (Hilden, Germany).

### Western Blot Assay

To confirm the presence and molecular mass of the MAGEA3 protein, Western blotting was performed using anti-His tag mAb. MAGE3 proteins were separated by 12% SDS-PAGE, transferred to a nitrocellulose membrane, and blocked with 5% nonfat milk in TBS at 37°C for 60 min. After washing with PBST, the membrane was incubated with 1:8,000 diluted anti-His mAb for 2 h. After washing, the filters were further incubated with 1:10,000 diluted HRP-conjugated goat anti-mouse IgG, and protein bands were visualized with 3,3′-diaminobenzidine tetrahydrochloride (DAB) kit according to the manufacturer’s instructions.

### Serum Specimens

The study included 93 GC patients, 107 chronic gastritis patients, and 108 healthy controls recruited from The First Affiliated Hospital of Wenzhou Medical University between February 2016 and December 2016. The three groups were matched for age and gender. Cancer patients who received radiation or chemotherapy before surgery were excluded. Histopathological data from surgical specimens were confirmed by the Department of Pathology. The study was approved by the Human Research Ethics Committee of The First Affiliated Hospital of Wenzhou Medical University, and written informed consent was obtained.

### ELISA Assay

Purified MAGEA3 protein and 1:200 diluted serum specimens were added to ELISA plates as first antibody; 1:10,000 diluted HRP-labeled human IgG was taken as secondary antibody. Three replicates were run for each sample. The results were quantified by recording the absorbance at 490 nm. Cutoff value = the mean A value of the healthy control + 3SD (33). Meanwhile, the study also analyzed the relationship between the positive rate of MAGEA3 antibodies in GC patients and clinicopathological features such as the TNM staging, pathological differentiation type, and transfer number of lymph nodes.

### Statistical Analysis

Statistical analyses were performed by R-3.5.3 and IBM SPSS Statistics 23. The experimental data were expressed as mean ± SD. Differences between two groups were assessed using the *t*-test. *χ* ^2^ test was performed to compare the difference of positive rate of MAGE-A3 antibodies in different groups.

## Results

### The mRNA Expression Level and Prognostic Value of MAGEA3 in Gastric Cancer

**Figure 1:**
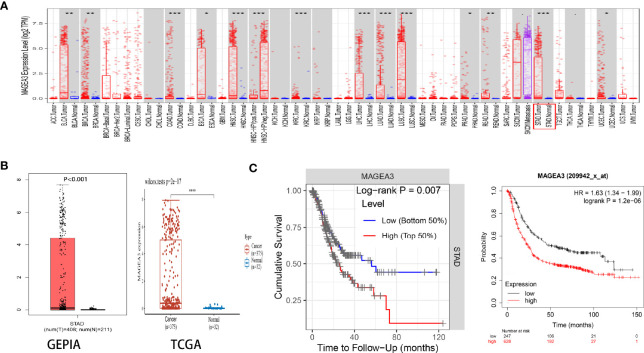
Expression of MAGEA3 in gastric cancer. **(A)** MAGEA3 expression in different types of cancer was investigated with the TIMER database. **(B)** Increased expression of MAGEA3 in gastric cancer compared to normal tissues in the GEPIA and TCGA database. **(C)** Correlation between MAGEA3 and prognosis of gastric cancer in TIMER and Kaplan-Meier plotter database. *p < 0.05; **p < 0.01; ***p < 0.001.

The mRNA expression of MAGEA3 in pan-cancer was first analyzed on the TIMER website. Higher expression of MAGEA3 was observed in GC (**Figure 1A**). A total of 32 normal controls and 375 gastric cancer patients were downloaded from TCGA database in March 2021. As shown in **Figure 1B**, MAGEA3 expression in the GC group is significantly higher than in the normal group (*p* < 0.01) in the TCGA. Similar upregulation of MAGEA3 expression was observed in GEPIA.

**Figure 2:**
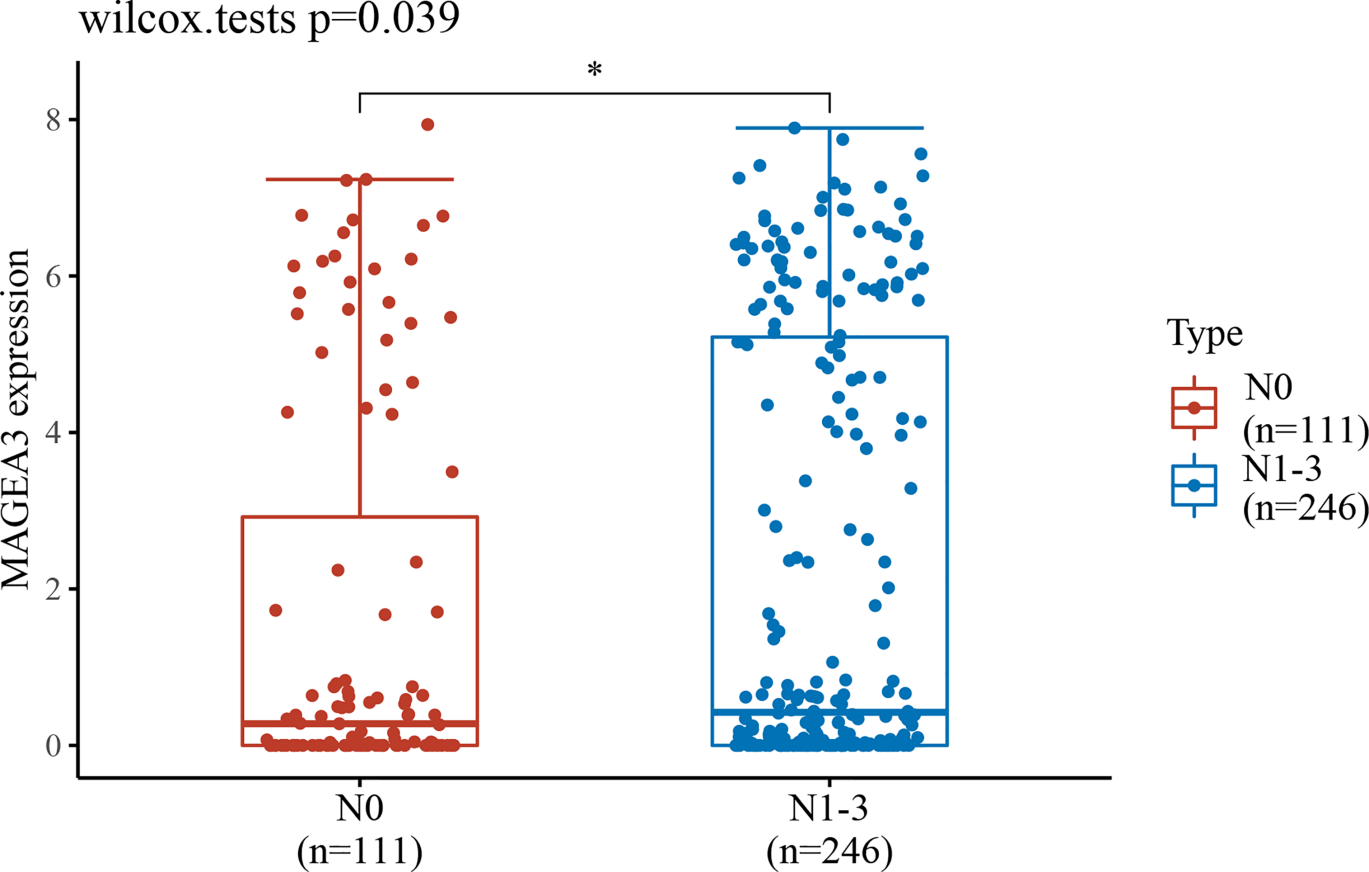
MAGEA3 expression increased significantly in gastric cancer–caused lymph node metastasis. *p < 0.05.

Since MAGEA3 is abnormally highly expressed in tumor sample, we subsequently investigated the influence of MAGEA3 expression on GC patients’ prognosis and clinicopathology. According to prognostic results from the TIMER database and Kaplan-Meier plotter database (**Figure 1C**), our results indicated that higher expression of MAGEA3 associated with poorer overall survival (OS) in GC patients (*p* < 0.05). We further evaluated the expression of MAGEA3 in GC patients with different N-stage. As shown in **Figure 2**, the expression of MAGEA3 in N+ was higher than that in N0 (*p* < 0.05), which may indicate that MAGEA3 expression is correlated to lymph node metastasis.

### Relationship Between MAGEA3 Expression and Tumor-Infiltrating Immune Cells

**Figure 3:**
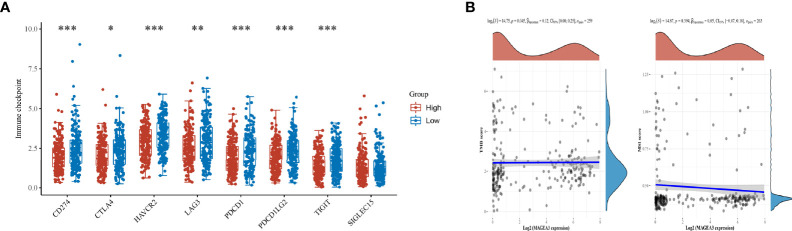
Differential expression of immune checkpoints inMAGEA3 groups and correlations of MAGEA3 expression with TMB and MSI. **(A)** The expression distribution of Immune checkpoints related gene in MAGEA3 high group and MAGEA3 low group. **(B)** Correlation analysis of MAGEA3 gene expression and TMB, MSI. *p < 0.05; **p < 0.01; ***p < 0.001.

**Figure 4:**
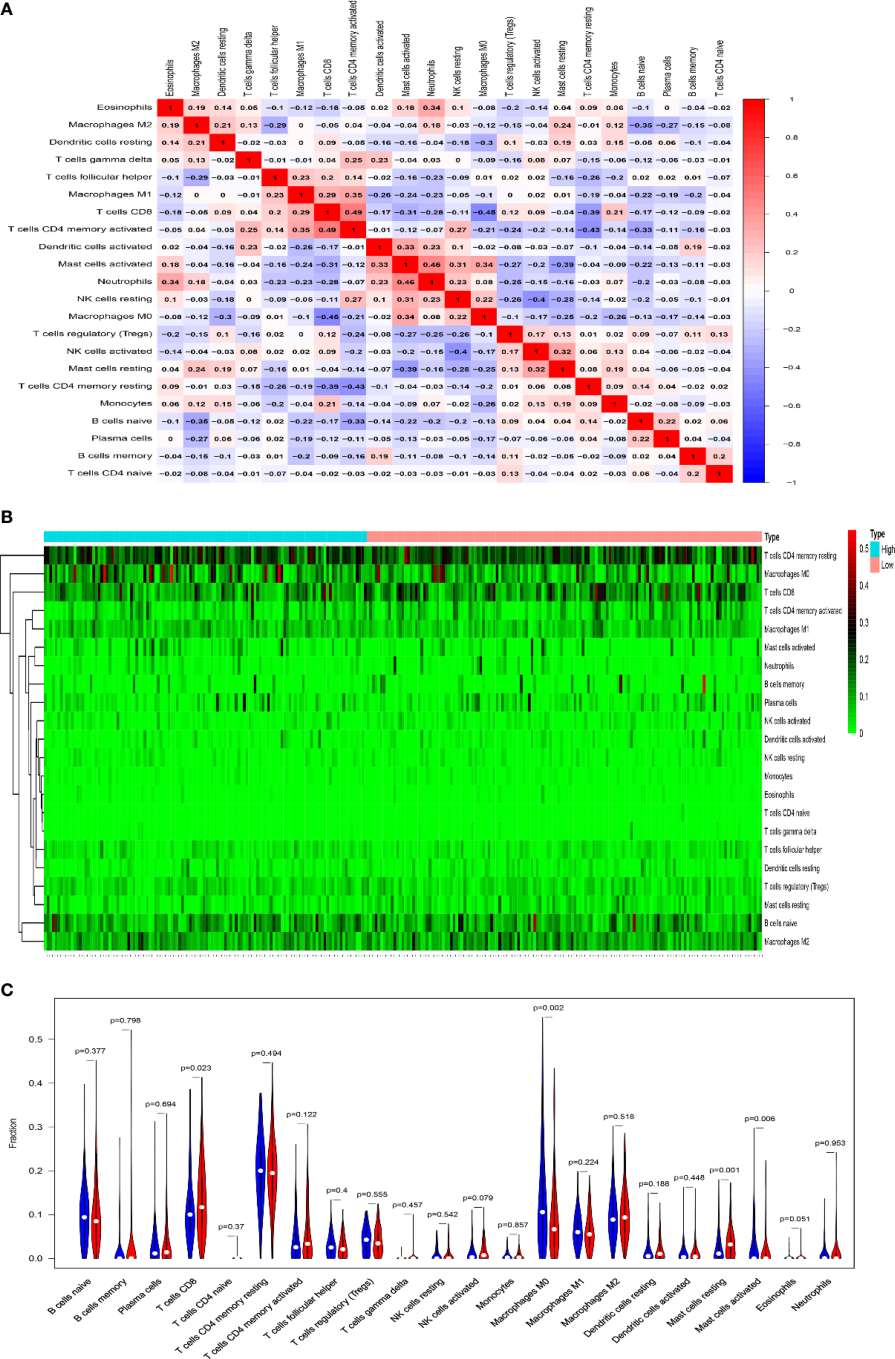
Results of CIBERSORT analysis and immune infiltration between MAGEA3 high- and low-expression groups. **(A)** Correlation matrix of infiltration degree of immune cells in GC samples. Red indicates trends consistent with the positive correlation, and blue indicates trends consistent with the negative correlation between two immune cells. The bigger size of the number statistics data represents the more positive or negative correlation. **(B)** The distribution of 22 immune cells in 267 filtered gene matrix. Red indicates higher immune infiltration expression, and green indicates lower expression. **(C)** Violin diagram of immune cell proportions in two groups. The blue fusiform fractions on the left represent the MAGEA3 high-expression group, and the red fusiform fractions on the right represent the MAGEA3 low-expression group.

Immune checkpoint inhibitors (ICI), a milestone in the field of cancer immunotherapy, has already improved treatment effect and survival in many cancer patients (34, 35). According to the expression level of MAGEA3, GC patients were divided into MAGEA3-high group (*n* = 188) and MAGEA3-low group (*n* = 187). Compared with the MAGEA3 low-expression group, the expression of immune checkpoint-related mRNA included CD274 (PD-L1), PDCD1 (PD-1), CTLA4, HAVCR2 (TIM-3), LAG3, PDCD1LG2 (PD-L2), and TIGIT, deregulated significantly (**Figure 3A**). We also found the expression of MAGEA3 correlates with TMB (**Figure 3B**), so we assume that the expression of MAGEA3 affects the immune status in gastric cancer. We further used CIBERSORT to explore the distribution of 22 types of immune cells in GC samples. The corHeatmap (**Figure 4A**) result showed that T-cell CD4 memory is activated and T-cell CD8 had a positive correlation (value = 0.49). T-cell CD8 had a negative correlation with macrophages M0 (value = −0.45). Correlation heatmap (**Supplementary Figure S1**) summarized the results obtained from 69 filtered gene expression matrix, and the relative percent of the 22 immune cells is shown in **Figure 4B**. Compared with the MAGEA3-low group, the violin plot of the immune cell showed that, macrophages M0, master cells activated infiltrated statistically more, while T-cell CD8, mast cells resting infiltrated statistically less (**Figure 4C**).

### MAGEA3 Expression Is Correlated With Immune Infiltration Level in GC in TIMER

**Figure 5:**
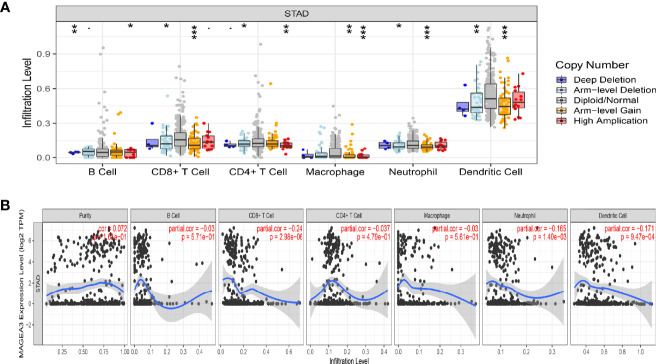
Correlation analysis of MAGEA3 expression and immune infiltration in GC by TIMER. **(A)** Association between MAGEA3 gene copy number and immune cell infiltration levels in GC cohorts. **(B)** Correlation of MAGEA3 expression with immune infiltration level in GC. *p < 0.05; **p < 0.01; ***p < 0.001.

Tumor-infiltrating lymphocytes (TILs) were viewed as a prognostic feature in many primary malignancies (36). In the TIMER database, the “SCNA” module results showed that altered MAGEA3 gene copy numbers seemed to associate with several immune cell infiltration levels, including CD8+ T cells, CD4+ T cells, B cells, dendritic cells (DCs), neutrophil, and macrophages in GC (**Figure 5A**). The “Gene” module results then showed that MAGEA3 expression has negative correlation with infiltration of CD8+ T cells (*R* = −0.24, *p* = 2.96e−06), neutrophil (*R* = −0.165, *p* = 1.4e−03). Dendritic cells (*R* = −0.171, *p* = 9.47e−07) (**Figure 5B**).

### The Association Between MAGEA3 and Immune Marker Expression

We explored the link between MAGEA3 expression and various immune markers in GC through the TIMER and TISIB databases, which included 28 TILs, immune stimulatory or inhibitory genes (including immune checkpoint gene sets), chemokine, chemokine receptors, and MHC genes (**Table 1**; **Supplementary Tables S1, S2**; **Supplementary Figure S2**). After adjustment by tumor purity, the analysis showed that the expression of MAGEA3 was significantly associated with most of the marker of immune cells in GC. The correlation between MAGEA3 and activated T-cells marker is listed in **Table 1**. In activated CD8 T cells, the correlation between 25 immune markers and the expression of MAGEA3 was analyzed. Interestingly, MAGEA3 expression was associated with 19 immune markers, except for ADRM1, CSE1L, and GEMIN6, which were positively correlated; the others are negatively correlated, including PIK3IP1 (*R* = −0.2510, *p* = 7.44e−07), which was recognized as a negative immunomodulator that inhibits antitumor T-cell immunity (37). For activated CD4 T cells, MAGEA3 expression was related to 17 of 25 immune markers, and most were negatively correlated, including TRAT1 (Trim), CCL5, ITK, etc. As shown in **Supplementary Figure S2**, **Supplementary Table S1**, MAGEA3 expression was associated with many types of TILs, and most of them were negatively correlated.

**Table 1:** Correlation analysis between MAGEA3 and markers of active T cells.

Activated CD8T cell	None		Purity		Activated CD4T cell	None		Purity	
	Cor	*p*	Cor	*p*		Cor	*p*	Cor	*p*
ADRM1	0.2151	9.81E−06^*^	0.2134	2.80E−05^*^	AIM2	**−**0.1683	5.76E−04^*^	**−**0.1559	2.33E−03^*^
AHSA1	0.0487	3.22E**−**01	0.0236	6.47E**−**01	BIRC3	**−**0.1500	2.19E−03^*^	**−**0.1222	1.73E−02^*^
C1GALT1C1	**−**0.0420	3.93E**−**01	**−**0.0450	3.82E**−**01	BRIP1	0.0227	6.45E**−**01	0.0118	8.19E**−**01
CCT6B	**−**0.1221	1.28E−02^*^	**−**0.1512	3.17E−03^*^	CCL20	0.0862	7.93E**−**02	0.0846	1.00E**−**01
CD37	**−**0.2260	3.31E**−**06^*^	**−**0.1959	1.24E−04^*^	CCL4	**−**0.1407	4.08E−03^*^	**−**0.1327	9.69E−03^*^
CD3D	**−**0.2298	2.23E−06^*^	**−**0.2170	2.04E−05^*^	CCL5	**−**0.2199	6.13E−06^*^	**−**0.2167	2.09E−05^*^
CD3E	**−**0.2333	1.55E−06^*^	**−**0.2165	2.13E−05^*^	CCNB1	0.1627	8.81E−04^*^	0.1552	2.44E−03^*^
CD69	**−**0.2366	1.09E−06^*^	**−**0.2209	1.43E−05^*^	CCR7	**−**0.1742	3.63E−04^*^	**−**0.1460	4.40E−03^*^
CD8A	**−**0.2190	6.72E−06^*^	**−**0.2129	2.94E−05^*^	DUSP2	**−**0.0556	2.58E**−**01	**−**0.0507	3.25E**−**01
CETN3	**−**0.0856	8.15E**−**02	**−**0.1082	3.52E−02^*^	ESCO2	0.0523	2.88E**−**01	0.0353	4.94E**−**01
CSE1L	0.3175	3.56E−11^*^	0.3064	1.11E−09^*^	ETS1	**−**0.1394	4.45E−03^*^	**−**0.1140	2.65E−02^*^
GEMIN6	0.1625	8.94E−04^*^	0.1543	2.60E−03^*^	EXO1	0.1758	3.19E−04^*^	0.1582	2.01E−03^*^
GNLY	**−**0.1425	3.64E−03^*^	**−**0.1423	5.50E−03^*^	EXOC6	**−**0.0361	4.63E**−**01	**−**0.0349	4.99E**−**01
GPT2	**−**0.0527	2.85E**−**01	**−**0.0681	1.86E**−**01	IARS	0.0160	7.45E**−**01	0.0011	9.83E**−**01
GZMA	**−**0.2400	7.52E−07^*^	**−**0.2412	2.03E−06^*^	ITK	**−**0.2202	5.94E−06^*^	**−**0.2049	5.83E−05^*^
GZMH	**−**0.1747	3.50E−04^*^	**−**0.1740	6.68E−04^*^	KIF11	0.1020	3.78E−02^*^	0.0796	1.22E**−**01
GZMK	**−**0.2182	7.28E−06^*^	**−**0.2066	5.06E−05^*^	KNTC1	0.1677	6.01E−04^*^	0.1702	8.78E−04^*^
IL2RB	**−**0.1969	5.38E−05^*^	**−**0.1848	2.99E−04^*^	NUF2	0.3041	2.49E−10^*^	0.2713	8.09E−08^*^
LCK	**−**0.1860	1.39E−04^*^	**−**0.1506	3.30E−03^*^	PRC1	0.2284	2.59E−06^*^	0.2157	2.29E−05^*^
MPZL1	**−**0.0172	7.27E**−**01	**−**0.0185	7.19E**−**01	PSAT1	0.0110	8.23E**−**01	0.0063	9.02E**−**01
NKG7	**−**0.2258	3.39E−06^*^	**−**0.2188	1.72E−05^*^	RGS1	**−**0.1542	1.63E−03^*^	**−**0.1409	5.99E−03^*^
PIK3IP1	**−**0.2699	2.32E−08^*^	**−**0.2510	7.44E−07^*^	RTKN2	0.1742	3.63E−04^*^	0.1622	1.53E−03^*^
PTRH2	0.0819	9.59E**−**02	0.0646	2.10E**−**01	SAMSN1	**−**0.1645	7.70E−04^*^	**−**0.1348	8.58E−03^*^
TIMM13	0.0041	9.33E**−**01	**−**0.0023	9.65E**−**01	SELL	**−**0.1406	4.10E−03^*^	**−**0.1017	4.80E−02^*^
ZAP70	**−**0.1872	1.25E−04^*^	**−**0.1739	6.73E−04^*^	TRAT1	**−**0.2129	1.22E−05^*^	**−**0.2031	6.83E−05^*^

Cor, R-value of Spearman’s correlation: *p < 0.05.

Bold means p < 0.05.

In **Supplementary Table S2**, the correlation between the expression of MAGEA3 and 44 common immune control genes was analyzed. The results showed that MAGEA3 expression was significantly associated with 34 immune checkpoint markers, including PDCD1 (PD-1), CD274 (PD-L1), CTLA4, etc. As we have known, CD274 (PD-L1), CTLA4, and PDCD1 (PD-1) were biomarkers of response to ICI and already used in cancer immunotherapy (38–40). Therefore, these results have proven that MAGEA3 may play a key role in tumor immunity. To further explain the effect of MAGEA3 expression on immune cell migration, we analyzed the relationship between MAGEA3 and chemokines and chemokine receptors. The results demonstrated that MAGEA3 expression was also associated with immune cell-associated chemokines and chemokine receptors, and most of them were negatively correlated, such as CCL5 (*R* = −0.2167, *p* = 2.09e−05), CXCR3 (*R* = −0.2274, *p* = 7.76e−06), and CXCL13 (*R* = −0.1707, *p* = 8.50e−04), those results may indicate that the expression of MAGEA3 may regulate the migration of immune cells to tumor microenvironment.

### Prognostic Analysis of MAGEA3 Expression in GC Based on Immune Cells

We have demonstrated that the expression of MAGEA3 was associated with the immune infiltration and prognosis in GC. Therefore, we inferred that the expression of MAGEA3 affected the prognosis, partly due to immune infiltration.

**Figure 6:**
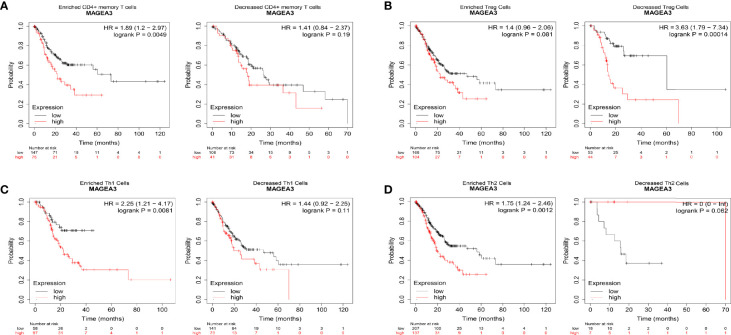
Comparison of Kaplan-Meier survival curves of the high and low expression of MAGEA3 in GC based on immune cell subgroups. Relationships between MAGEA3 of different immune cell subgroup and prognoses in gastric cancer **(A**–**D)**.

We did a prognosis analysis based on the MAGEA3 expression levels in the relevant immune cell subgroups *via* the Kaplan Meier plotter. The results showed that the low expression of MAGEA3 in GC in enriched CD4 memory T cells, enriched Th1 cells, and enriched Th2 cell cohort had better prognosis respectively, but there was no significant correlation in decreased immune cell groups (**Figures 6A, C, D** **)**. On the contrary, we found that the low expression of MAGEA3 in decreased Treg cell cohort had a better prognosis (**Figure 6B**). The above analysis indicated that the MAGEA3 expression in GC may affect prognosis partly because of immune infiltration.

### Gene Sets Enriched Analysis About MAGEA3 in Gastric Cancer

**Figure 7:**
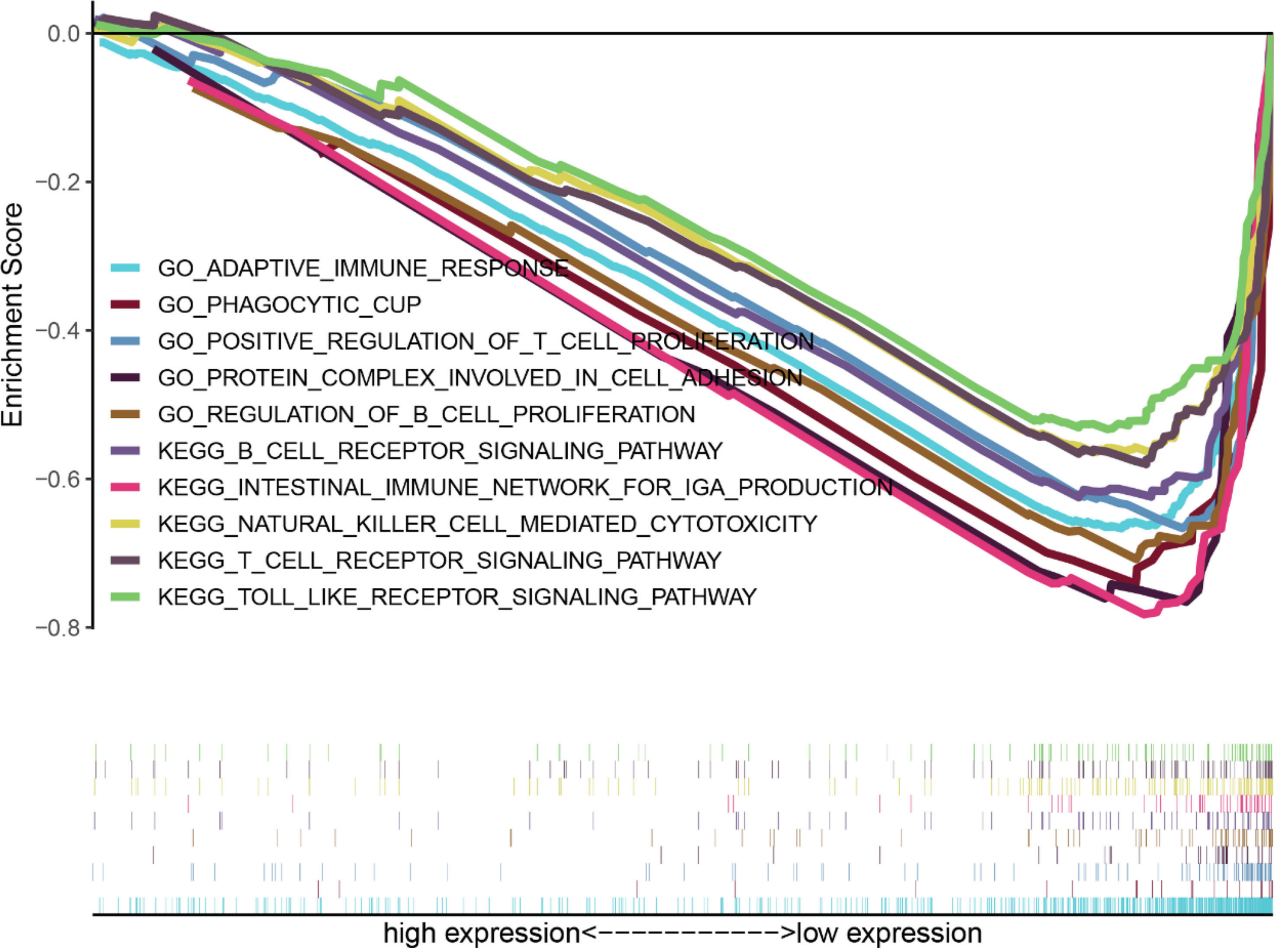
Enrichment plots from gene set enrichment analysis (GSEA).

MAGEA3-related signaling pathways involved in GC between low and high MAGEA3 expression were identified through GSEA and demonstrated significant differences (NOM *p*-value <0.05 and FDR <0.05, |NES|>1) in enrichment of GO and KEGG collection. We only listed 5 pathways of GO and KEGG because of limited space (**Table 2**). As shown in **Figure 7**, 5 KEGG items including intestinal immune network for IgA production, B-cell receptor signaling pathway, T-cell receptor signaling, natural killer cell-mediated cytotoxicity, and toll-like receptor signaling pathway were enriched in MAGEA3 low-expression phenotype. Five GO items including regulation of B-cell proliferation, protein complex involved in cell adhesion, adaptive immune response, positive regulation of T-cell proliferation, and phagocytic cup have shown significant differential enrichment in MAGEA3 low-expression phenotype. There are no KEGG or GO items enriched in MAGEA3 high-expression phenotype based on NES, NOM *p*-value, and FDR value. These all suggest that MAGEA3 plays an immunomodulatory role in gastric cancer.

**Table 2:** Gene sets enriched in phenotype.

Gene set name	NES	NOM *p*-val	FDR *q*-val
KEGG_INTESTINAL_IMMUNE_NETWORK_FOR_IGA_PRODUCTION	−2.11	0	0.012
KEGG_NATURAL_KILLER_CELL_MEDIATED_CYTOTOXICITY	−1.97	0.002	0.031
KEGG_B_CELL_RECEPTOR_SIGNALING_PATHWAY	−1.92	0.006	0.031
KEGG_T_CELL_RECEPTOR_SIGNALING_PATHWAY	−1.89	0.004	0.033
KEGG_TOLL_LIKE_RECEPTOR_SIGNALING_PATHWAY	−1.84	0.01	0.045
GO_REGULATION_OF_B_CELL_PROLIFERATION	−2.17	0	0.048
GO_PROTEIN_COMPLEX_INVOLVED_IN_CELL_ADHESION	−2.15	0	0.0034
GO_ADAPTIVE_IMMUNE_RESPONSE	−2.13	0.004	0.044
GO_PHAGOCYTIC_CUP	−2.12	0	0.0039
GO_POSITIVE_REGULATION_OF_T_CELL_PROLIFERATION	−2.12	0	0.048

### PPI Network Construction of MAGEA3-Related Partners

**Figure 8:**
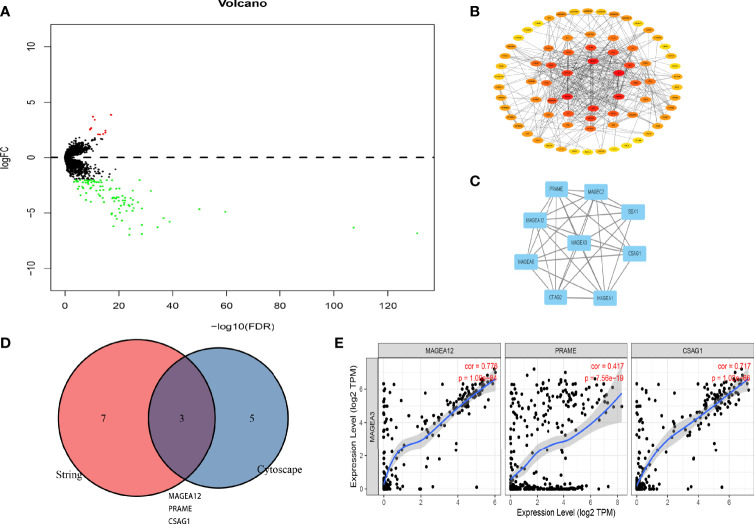
PPI network analysis of MAGEA3-related genes. **(A)** Volcano plot of the differentially expressed genes between MAGEA3 high group and MAGEA3 low group. **(B)** PPI structureof co-expressed genes. **(C)** The top module involved MAGEA3 identified by MCODE plugin in Cytoscape. **(D)** An intersection analysis of the MAGEA3-binding and correlated genes was conducted. **(E)** Correlation analysis between MAGEA3 expressed and screened common genes, including MAGEA12, PRAME, and CSAG1.

DEGs in MAGEA3-high group and MAGEA3-low group were analyzed by “Limma” package. As shown in **Figure 8A** and **Supplementary Figure S3**, 11 significantly upregulated genes and 97 significantly downregulated genes were identified. We also performed a series of enrichment analyses based on these DEGs, including KEGG and GO (**Supplementary Figure S4**). To understand potential interactions among these DEGs, we also performed a PPI network analysis by utilizing STRING and Cytoscape. We first constructed the MAGEA3 coexpression gene network through Cytoscape (**Figure 8B**), then filtered out the module that MAGEA3 was involved in *via* the MCODE plugin (**Figure 8C**), and screened out eight genes interacting with MAGEA3 including MAGEA12, MAGEA6, CTAG2, MAGEA1, CSAG1, SSX1, MAGEC2, and PRAME. We also listed 10 MAGEA3-binding proteins with the experimental evidence identification based on the STRING database (**Supplementary Figure S5**). A Venn analysis showed three common members: MAGEA12, PRAME, and CSAG1. MAGEA3 expression was positively correlated with that of MAGEA12 (*R* = 0.776, *p* = 1.09e−84), PRAME (*R* = 0.417, *p* = 7.56e−19), and CSAG1 (*R* = 0.717, *p* = 1.02e−66) in GC (**Figures 8D, E**).

### Prediction and Analysis of Potential miRNAs Associated With MAGEA3

**Figure 9:**
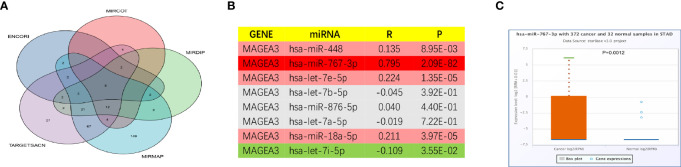
Prediction potential miRNA of MAGEA3 in GC. **(A)** The predicted miRNAs targeting MAGEA3 in five databases. **(B)** The expression correlation between predicted miRNAs and MAGEA3 in GC analyzed by starBase database. **(C)** The expression of hsa-miR-767-3p in GC and control normal samples determined by starBase database.

As we have known, microRNAs are involved in the regulation of gene expression. We first predicted miRNAs that could potentially bind to MAGEA3 and finally found 8 miRNAs targeting MAGEA3 from TargetScan (http://www.targetscan.org/vert_71/), mirDIP (http://ophid.utoronto.ca/mirDIP/), ENCORI website [ENCORI: The Encyclopedia of RNA Interactomes. (sysu.edu.cn)], microT [DIANA TOOLS - microT-CDS (athena-innovation.gr)], and miRmap [miRmap (ezlab.org)] (**Figure 9A**). As listed in **Figure 9B**, MAGEA3 was negatively correlated with hsa-let-7i-5p and positively correlated with hsa-miR-448, hsa-miR-767-3p, hsa-let-7e-5p, and hsa-miR-18a-5p in GC. Moreover, hsa-miR-767-3p exhibited the strongest correlation with MAGEA3 (*R* = 0.795, *p* = 2.09e−82), and the expression of hsa-miR-767-3p was significantly different in gastric cancer and normal tissues (**Figure 9C**).

### Production of the MAGEA3 Full-Length Protein

**Figure 10:**
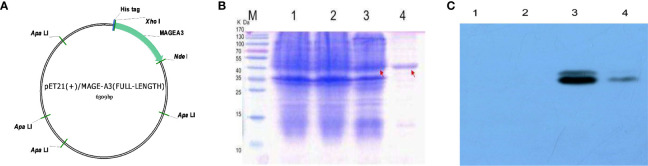
pET21(+)/MAGEA3 plasmid contruction and purification. **(A)** The map of the expression vector pET21a(+)/MAGEA3 codon-optimized MAGEA3 942bp with 6xHis tag cloned into the prokaryotic expression vector pET21a(+) by NedI/XhoI restriction sites. **(B)** Affinity purified MAGE-A3 protein expressed from E. coli Rosetta were analyzed with SDS-PAGE. **(C)** Affinity purified MAGE-A3 identified by western blot with mouse anti-His mAb. Lane M: pre-stained protein marker; Lane 1, Rosetta; Lane 2, pET21a(+)/Rosetta; Lane 3, pET21a(+)/MAGE-A3 at 48 kDa; Lane4, purified MAGE-A3protein at 48 kDa.

The codon-optimized MAGEA3 digested with restriction endonucleases *Ned*I and *Xho*I was inserted into the pET21a (+) vector (**Figure 10A**). The recombinant plasmid was then transform into *E. coli* Rosetta and induced by IPTG. **Figure 10B** shows a band at an appropriate position around 48 kD, corresponding to MAGEA3 protein. The protein was then subjected to Western blot with the antibody against His-tag to identity its specificity. The purified MAGEA3 protein (250 µg/ml) was obtained through Ni–NTA agarose affinity chromatography (**Figure 10C**). This study also conducted the immunogenicity and antigenicity analysis of the MAGEA3 protein (**Supplementary Figures S6–S8**).

### Serum Detection of MAGEA3 Antibodies in GC Patients

**Figure 11:**
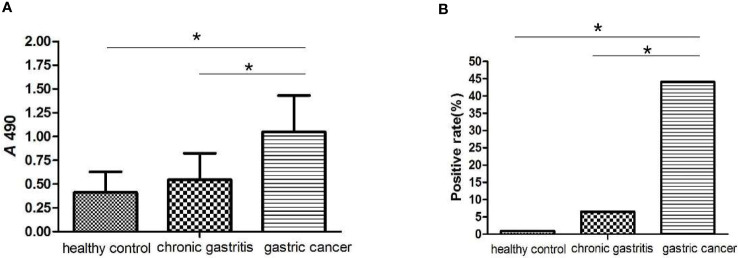
ELISA analysis of MAGE-A3 specific IgG antibodies in the serum of gastric cancer. **(A)** MAGE-A3 specific serum IgG antibody levels in three groups of patients with healthy controls, chronic gastritis and gastric cancer. **(B)** The serum antibody positive detection rate of MAGE-A3 in each group. *P<0.05.

The MAGEA3-specific IgG was detected in 93 GC patients, 107 chronic gastritis patients, and 108 healthy controls by ELISA. As shown in **Figure 11A**, serum concentration of MAGEA3 antibodies in the GC group (1.049 ± 0.384) was significantly higher than that of patients with chronic gastritis (0.546 ± 0.278) and healthy controls (0.412 ± 0.218) (F1 = 15.096, *p* < 0.01; F2 = 34.373, *p* < 0.01). The cutoff value was calculated as 1.065, which was further applied to calculate the positive rate of MAGEA3-specific IgG. The results show (**Figure 11B**) that the positive rate in GC was 44.08% (41/93) and gastritis and healthy control groups was 6.54% (7/107) and 0.92% (1/109), respectively. The positive rate of serum antibody in the GC group was significantly higher than the other two groups ( $\chi_{1}^{2}$ = 38.450, *p* < 0.01; $\chi_{2}^{2}$ = 56.082, *p* < 0.01). Regardless of serum concentration or positive rate, there was no statistically significant differences between gastritis and healthy control groups (*p* > 0.05). The sensitivity of ELISA to detect MAGEA3-specific IgG for serological diagnosis of GC was 44.08%, while the specificity was 92.54%.

### Correlation Between MAGEA3 and Clinical Analysis

The research obtained 43 patients’ histopathological data among 93 gastric cancer patients who underwent radical surgery (**Table 3**). No significant differences were identified between the positive rate of MAGEA3 antibodies and pathological differentiation type, diameter of tumor, and patient age and gender. However, the positive rate of serum MAGEA3 antibodies in stages III and IV was significantly higher than the rate in stages I and II (*p* < 0.05). The positive rate in more lymph node metastasis (*N* ≥ 3) was also significantly higher than that in the less lymph node metastasis (*N* = 0–2).

**Table 3:** Clinicopathological features and MAGEA3 antibodies detection of 43 patients.

Clinicopathological features	Positive rate of MAGEA3 antibodies (Positive cases/total number)	*p*
**Gender**		0.207
Male	0.46 (13/28)	
Female	0.27 (4/15)	
**Age (years)**		0.559
<60	0.46 (6/13)	
≥60	0.37 (11/30)	
**Tumor size (cm)**		0.350
<5	0.33 (8/24)	
≥5	0.47 (9/19)	
**Differentiation**		0.181
Well/moderately	0.28 (5/18)	
Poorly/none	0.48 (12/25)	
**Transfer of lymph node(N)**		0.021^*^
0–2	0.28 (8/29)	
≥3	0.64 (9/14)	
**TNM stage**		0.001^*^
I/II	0.18 (5/28)	
III/IV	0.80 (12/15)	

TNM, tumor node metastasis. ^*^p < 0.05.

## Discussion

Although rapid clinical treatment progress has changed survival of gastric cancer beyond recognition, the prognosis of GC patients still remains unsatisfactory. Immunotherapy such as anti-PD-1 or anti-PD-L1 therapy is of paramount importance to advanced GC patients (2). Despite Epstein-Barr virus (EBV) status, PD-L1 and MSI serve as predictive markers; poor response or developing resistance is quite common in many GC patients when receiving immunotherapy (12). Hence, we need to figure out the mechanism of those limitations and develop strategies that could improve sensitivity to immunotherapy in GC patients. Meanwhile, biomarkers that could predict the prognosis of GC patients are at the forefront of recent studies, which inspire us to find a new biomarker with therapeutic value in GC patients (11, 41).

Exactly like numerous proteins belong to MAGE gene family, MAGEA3 could bind with E3 Really Interesting New Gene (RING) and then enhance its ubiquitin ligase activity (42, 43). Even though the expression of MAGEA3 is generally confined to germline cells of the testis and placenta the same as other CTA, MAGEA3 may function as a potential immunotherapeutic target with its elevated expression in diverse malignant tumor cells including melanoma (19), lung cancer (20), and colorectal cancer (21). Many clinical trials with this antigen also have been carried out, which include being vaccinated with a strictly tumor-specific MAGEA3 peptide or MAGEA3 protein. MAGE-3 peptide vaccine exhibited therapeutic benefits and improved disease-free survival in melanoma and lung cancer patients according to preceding clinical trials (44–46). Our group also identified three predictive immunodominant MAGEA3 epitopes that could provoke a high concentration of IgG targeting MAGEA3 in mice (28, 29). However, on account of the capability to trigger strong T-cell responses, MAGEA3 protein induced by recombinant technology becomes all the rage in abundant patients with MAGEA3-elevated tumors (47). The data from the phase 2 randomized NSCLC trial and patients with melanoma encouraged and moved the MAGEA3 immunotherapeutic forward (48, 49), while DERMA and MAGRIT which are phase III clinical trials indicated that MAGEA3 immunotherapeutic did not benefit overall survival or disease-free survival of patients with NSCLC or melanoma (26, 27). Sometimes cancer patients with MAGEA3 mRNA positive still manifest MAGEA3 immunotherapy resistance because no functional protein was produced at all (27). Another possible explanation is deficiency of T-cell responses (especially CD8 responses) contributing to the absence of clinical effects (50). So far, there is still no research about what role MAGEA3 plays in the immunotherapy of GC.

In our research, its biological roles and possible mechanism in GC were investigated by thorough bioinformatics analysis. We first discovered that MAGEA3 was elevated in tumor tissues, which was significantly correlated with poor OS. Simultaneously, high MAGEA3 expression was linked to the lymph node metastasis of gastric cancer, which was verified in subsequent serological studies. This phenomenon is similar to the previous research results. Futawatari et al. (51) reported the elevated expression rate of MAGEA3 was found in cancer patients with lymph node metastasis and venous invasion compared with those without. Honda et al. (14) found that 66% of GC patients studied exposed MAGEA3 hypomethylation, which is positively correlated with lymph node metastasis.

ICI play a crucial role in immunotherapy (52). Immunotherapy that uses ICI such as nivolumab and pembrolizumab to inhibit PD-1/PD-L1 axis has been the vogue for advanced GC patients (53–55). Cytotoxic T-lymphocyte-associated protein (CTLA)4 was also a receptor that attenuates the T-cell response. Approved by the U.S. Food and Drug Administration (FDA), ipilimumab is the first CTLA4 inhibitor that could enhance anticancer immunity (56). TIM3 acts as a “checkpoint” receptor, inhibiting TIM3, which can enhance the antitumor effect of PD1 blockade (57). In this study, we unveiled PD1, PD-L1, PD-L2, CTLA4, TIGIT, TIM-3, and LAG3 were deregulated expression significantly in the MAGEA3 high group, and the expression of MAGEA3 negatively correlates with TMB. As we have known, TMB affects sensitivity to immunotherapy using checkpoint inhibitor by regulating the production of immunogenic peptides (58). The above results may explain a surprising relationship between increased expression of a particular subset of MAGEA antigens (include MAGEA3) and poor ICI response (59).

An additional key finding in this study is that the expression of MAGEA3 correlated with the degree of immune infiltration in GC. Using CIBERSORT analytical tool, we found that macrophages M0, master cells activated were higher in the MAGEA3 high-expression group, while T-cell CD8, mast cells resting were higher in the MAGEA3 low-expression group. We also found that MAGEA3 expression has a negative correlation with infiltration of CD8+ T cells, neutrophil, and dendritic cells through the TIMER database analysis. Moreover, MAGEA3 was significantly correlated with most immune marker sets of various immune cells in GC. These findings together indicate that MAGEA3 may have an impact on the changes of tumor immune microenvironment. Prognostic analysis of MAGEA3 expression levels in different tumors based on immune cells was performed; high MAGEA3 expression level in GC had a poor prognosis in the enriched CD4 + memory T cell, enriched type 1 T helper cell, and enriched type 2 T helper cell subgroups. Thus, high expression of MAGEA3 in GC may affect the prognosis of GC patients in part due to immune infiltration.

We also performed GSEA to further study the functions of MAGEA3 in GC. GSEA showed that regulation of B-cell proliferation, adaptive immune response, and positive regulation of T-cell proliferation in GO were differentially enriched in MAGEA3 low-expression phenotype. Intestinal immune network for IgA production, B-cell receptor signaling pathway, T-cell receptor signaling, natural killer cell-mediated cytotoxicity, and toll-like receptor signaling pathway in KEGG were differentially enriched in MAGEA3 low-expression phenotype. These results indicated that MAGEA3 high expression results in immune suppression in GC.

Previous studies have shown that promoter demethylation and histone acetylation mediate the MAGEA3 gene expression (60, 61). Several studies have reported the expression rate of MAGEA3 in GC, but the range was wide, varying from 35% to 45%, and most of them were evaluated at mRNA level (51, 62). In the present study, we prepared and expressed MAGEA3 proteins by the prokaryotic expression system. When MAGEA3 protein was used as an ELISA diagnostic antigen, it can be recognized by tumor that expressed MAGEA3. The positive rate was 44.08%, which agree with previously reported rates, were obviously higher than control groups. Full-length MAGEA3 protein also contain epitopes for both T cells that carry the CD4 or CD8 antigen that have been detected in people with cancer; these epitopes can generate protective immunity through binding MHC class I or MHC class II molecules (63, 64), so injected MAGEA3 protein can induce the high-level humoral and cellular immune responses, and a large proportion of gastric cancer patients who were MAGEA3 positive identified by ELISA may be candidates for immunotherapy. MAGEA3 may be used not only as a diagnostic agent for gastric cancer patients but also as a potential target antigen for gastric cancer immunotherapy.

In summary, our study revealed that MAGEA3 is associated with lymph node metastasis and correlates with immune infiltration levels in GC. We also screened out MAGEA3 interacting proteins and miRNA which need further experimental validation. We believe MAGEA3 can serve as a new biomarker in gastric cancer and provide more effective therapies in the era of immunotherapy.

## Data Availability Statement

The datasets presented in this study can be found in online repositories. The names of the repository/repositories and accession number(s) can be found in the article/**Supplementary Material**.

## Ethics Statement

The studies involving human participants were reviewed and approved by the Ethics Committee of The First Affiliated Hospital of Wenzhou Medical University. The patients/participants provided their written informed consent to participate in this study. The animal study was reviewed and approved by the Ethics Committee of The First Affiliated Hospital of Wenzhou Medical University. Written informed consent was obtained from the individual(s) for the publication of any potentially identifiable images or data included in this article.

## Author Contributions

JJ and JT: conceptualization, methodology, and writing—original draft. JR and YC: software and visualization. WC: complete basic experiments and verification. LZ: analyzed and interpreted the data. QZ: review and editing. GZ: project administration. All authors contributed to the article and approved the submitted version.

## Funding

This study was supported by grants from The Public Welfare Foundation of Zhejiang Province (No. Lgf18h160030) and the Wenzhou Basic Scientific Research Projects (No. Y20210941).

## Conflict of Interest

The authors declare that the research was conducted in the absence of any commercial or financial relationships that could be construed as a potential conflict of interest.

## Publisher’s Note

All claims expressed in this article are solely those of the authors and do not necessarily represent those of their affiliated organizations, or those of the publisher, the editors and the reviewers. Any product that may be evaluated in this article, or claim that may be made by its manufacturer, is not guaranteed or endorsed by the publisher.
