# Supplementary material for: Comprehensive Analysis to Identify MAGEA3 Expression Correlated With Immune Infiltrates and Lymph Node Metastasis in Gastric Cancer
Source: Front Oncol. 2021 Dec 14;11:784925. doi: 10.3389/fonc.2021.784925 (PMC8712941; doi:10.3389/fonc.2021.784925)
Supplement: Supplementary file 1 [file DataSheet_1.docx]

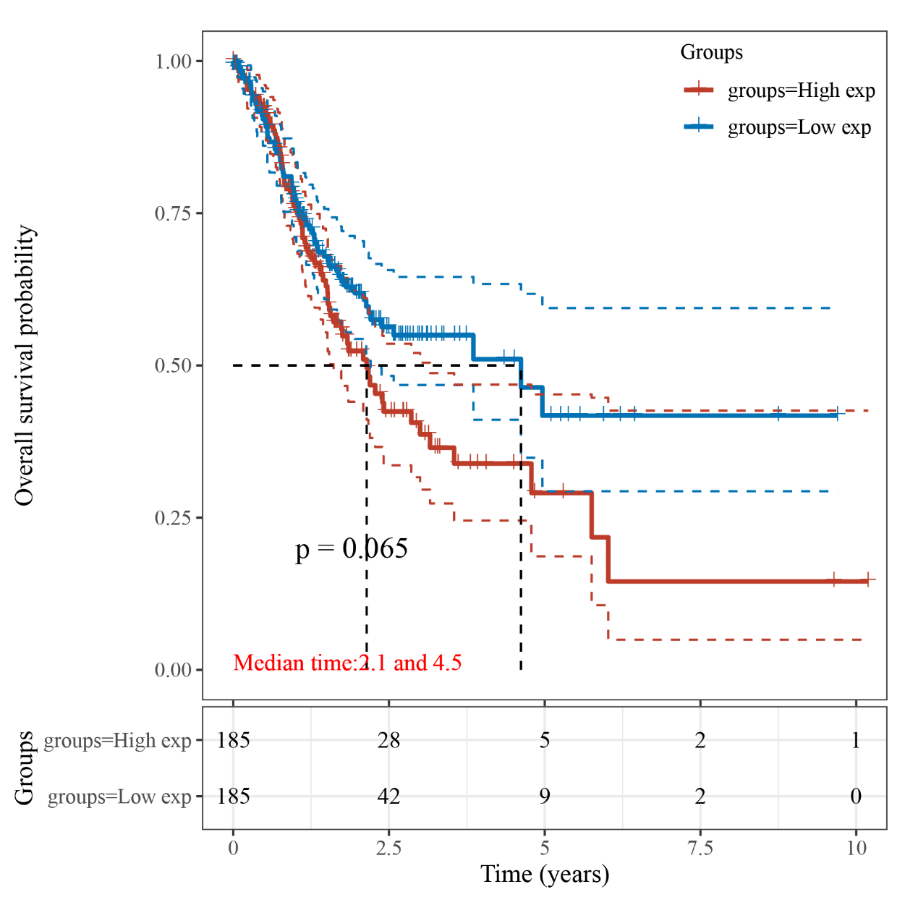


Fig.S1. Low and high expression of MAGEA3 were compared using a Kaplan-Meier survival curve based on TCGA data.


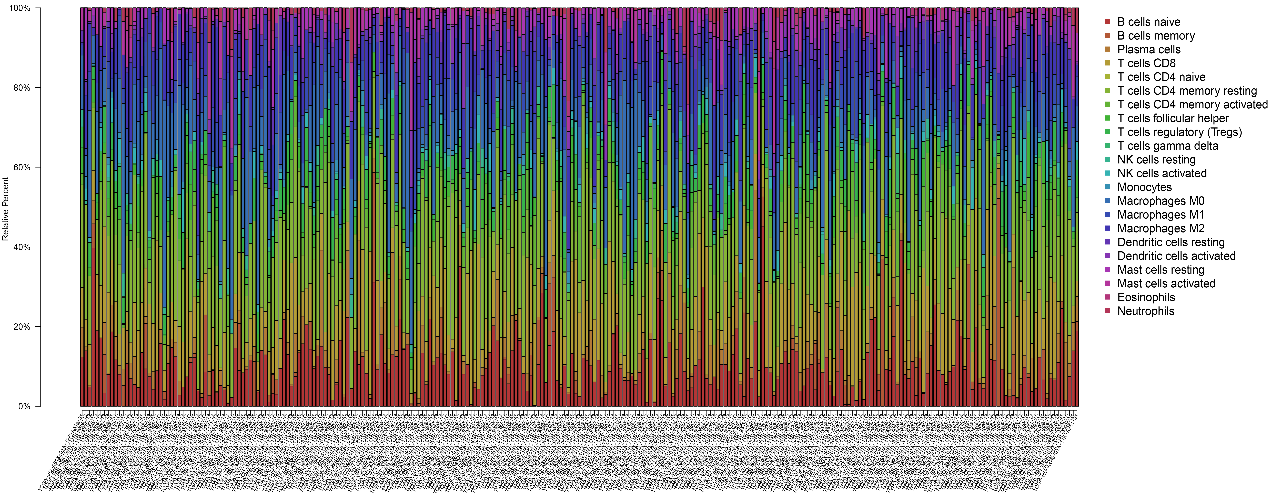


Fig.S2. Landscape of immune cell infiltration


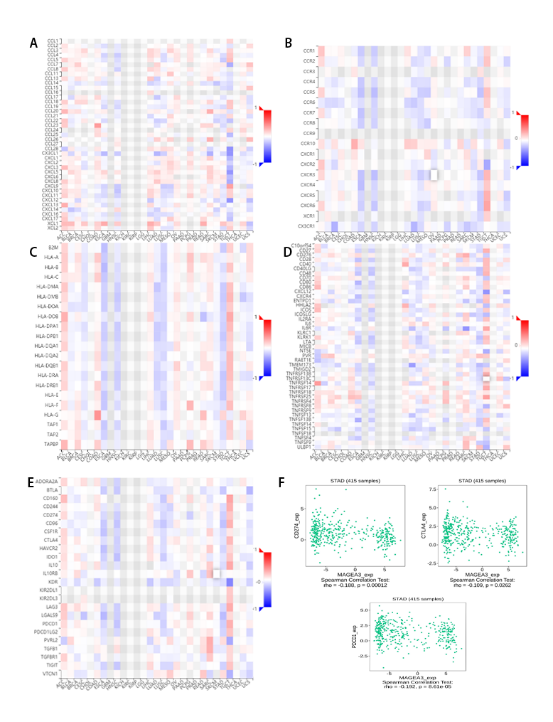


Fig.S3. A correlation between the expression of the MAGEA3 and genes encoding immune regulatory molecules in TISIB database. (A). The landscape of relationship between MAGEA3 expression and Chemokine in multiple types of cancers. (B). The landscape of relationship between MAGEA3 expression and Reception in multiple types of cancers. (C). The landscape of relationship between MAGEA3 expression and MHC in multiple types of cancers. (D). The landscape of relationship between MAGEA3 expression and Immunostimulator in multiple types of cancers. (E). The landscape of relationship between MAGEA3 expression and Immunoinhibitor in multiple types of cancers. (F) TPM4 expression was significantly negatively associated with CD274(PD-1), CTLA4, PDCD1(pd-L1).


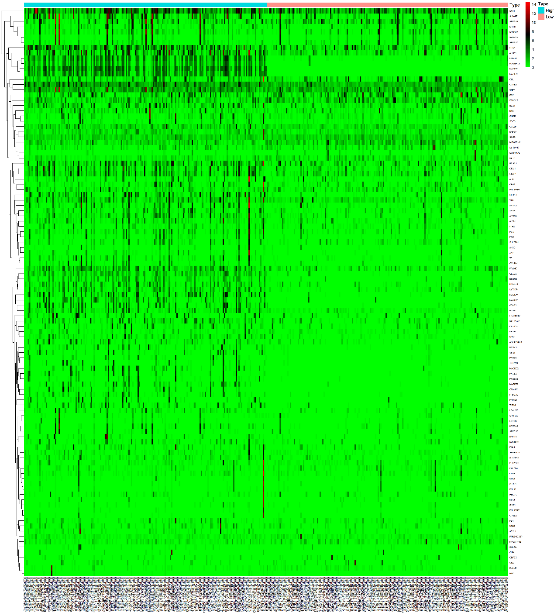


Fig.S4 Heatmap of DEGs according to the adjusted P-value and logFC. Red indicates higher gene expression and green indicates lower gene expression.


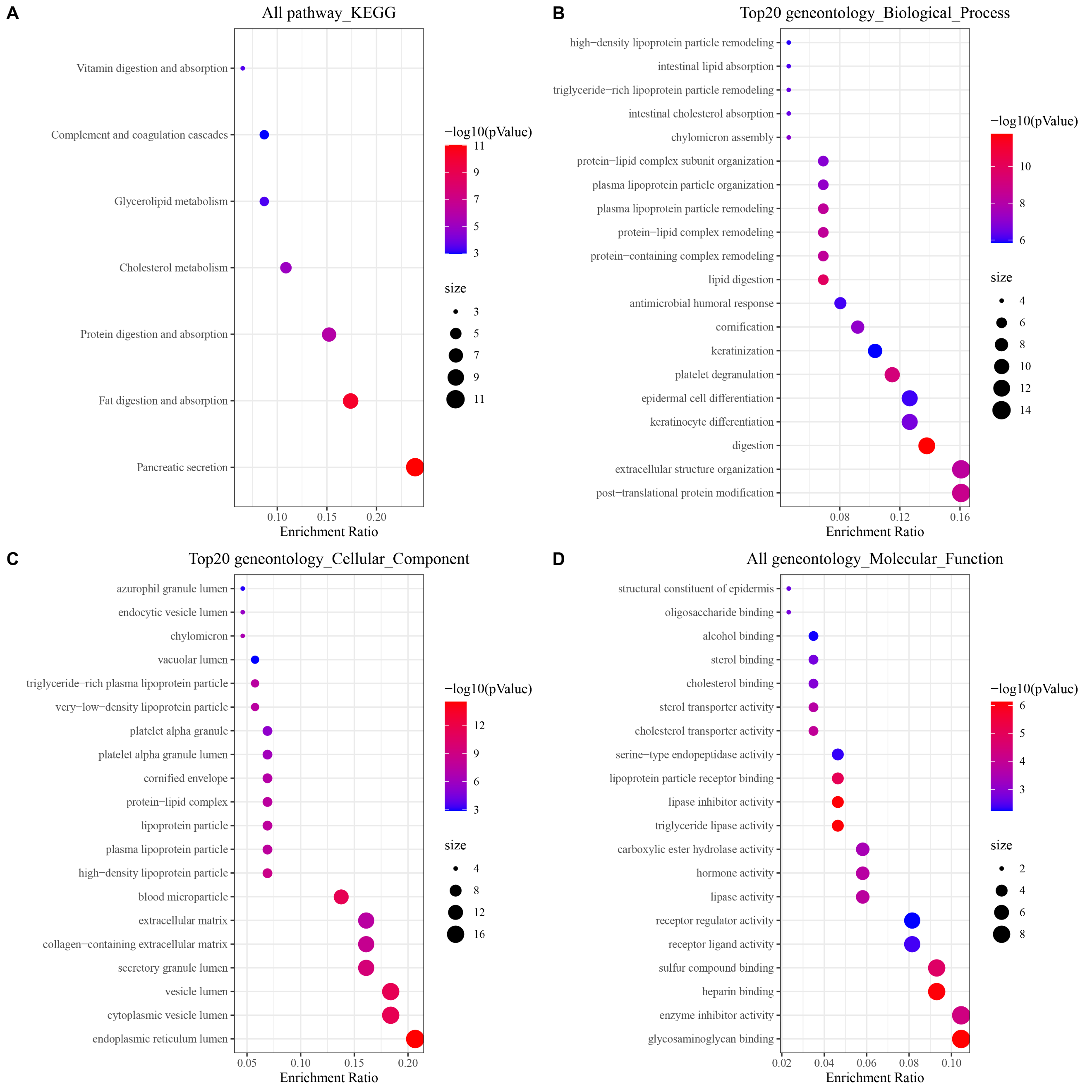


Fig.S5. GO and KEGG pathway enrichment analysis of DEGs.


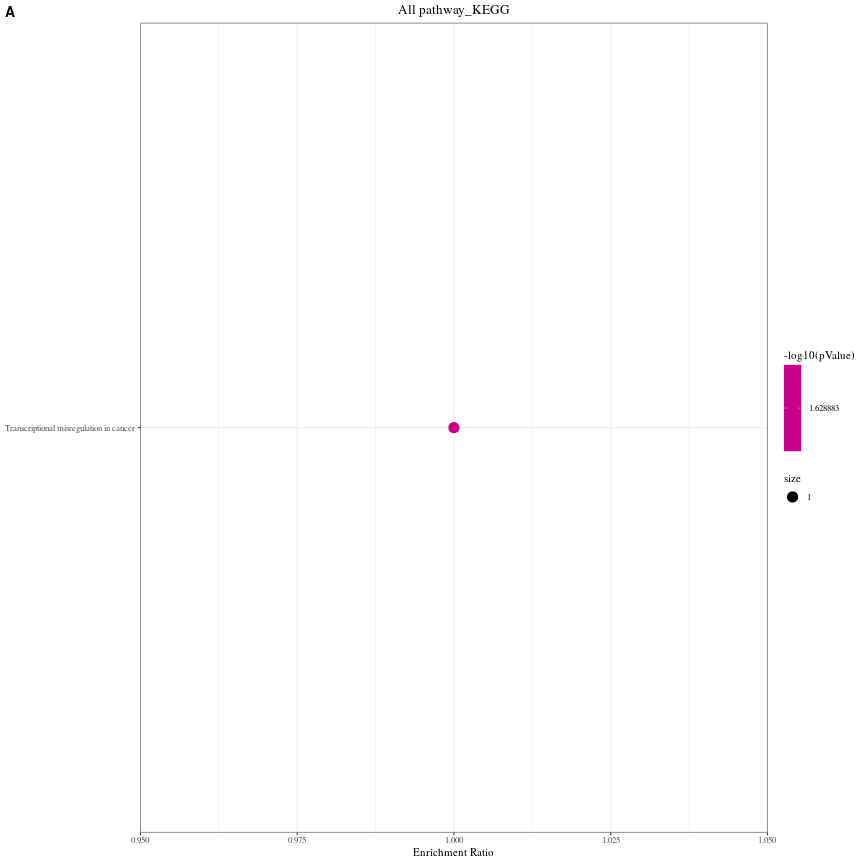


Fig.S6. KEGG pathway analysis of genes in the top module involved MAGEA3.


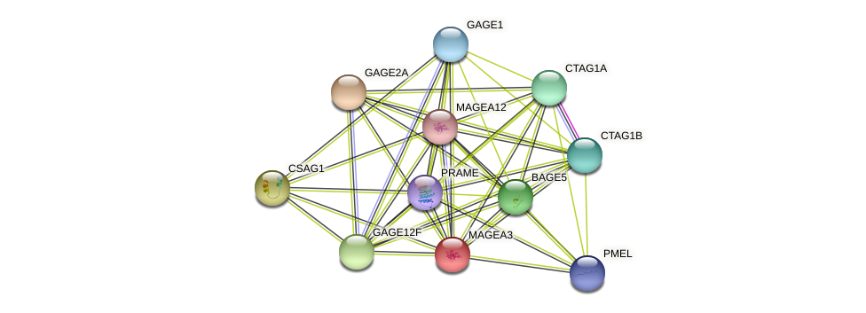


Fig.S7. The visualizing interaction network of MAGEA3-binding proteins was obtained base on STRING database.


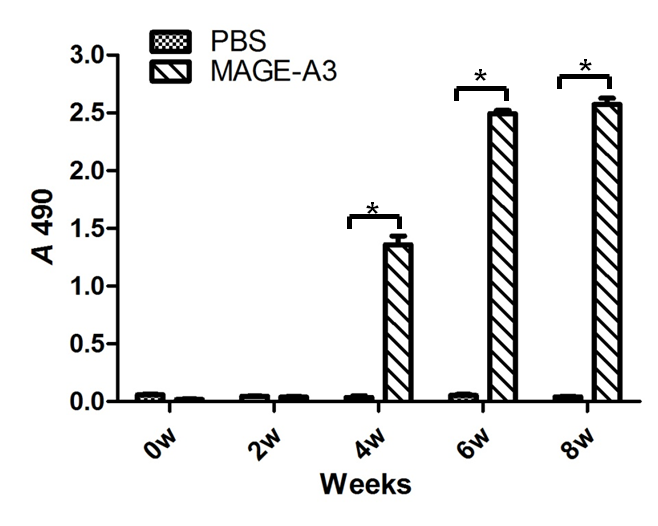


Fig.S8 Detection of antibodies response in mice to MAGE-A3 in different time by ELISA BALB/c mice were immunized with purified MAGE-A3 protein or PBS and collected their serum for determination of IgG antibodies against MAGE-A3 following on 0,2,4,6,8weeks. The results showed that MAGE-A3 specific antibody increased following the immune weeks, and remain high level at least until week 8. *P<0.05


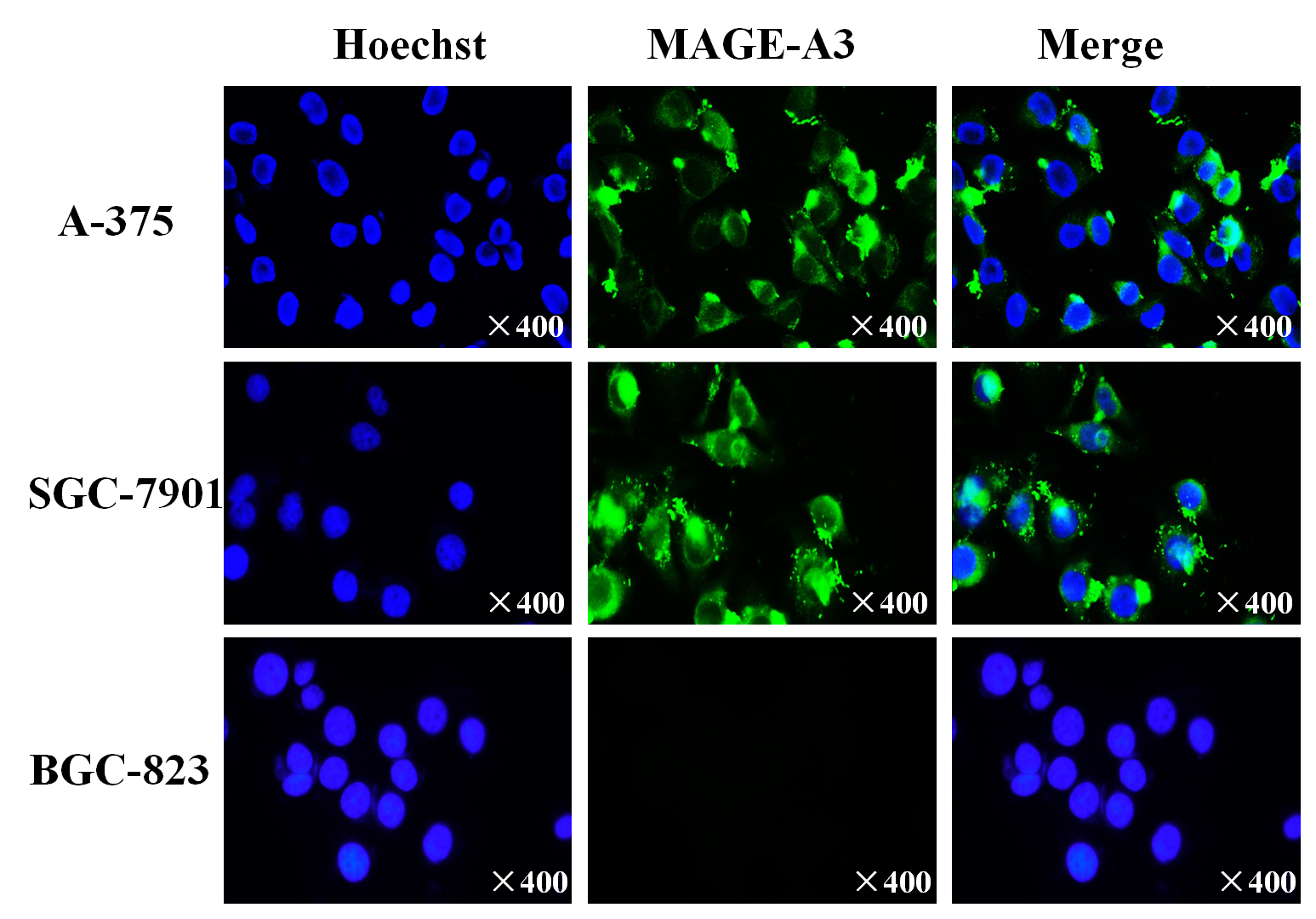


Fig.S9 IFA analysis of immune sera of MAGE-A3 protein recognized by native antigen. The recognition of the specific antibodies induced by the immune sera of MAGE-A3 protein to intracellular regions of human malignant melanoma cell line A375, Gastric cancer cell line SGC-7901 and BGC-823 were analyzed by IFA. The A735 cells, SGC-7901 cells' cytoplasm could recognize the MAGE-A3 antibodies and emitted green fluorescence, indicated the MAGE-A3 protein is located primarily in the cells' cytoplasm.


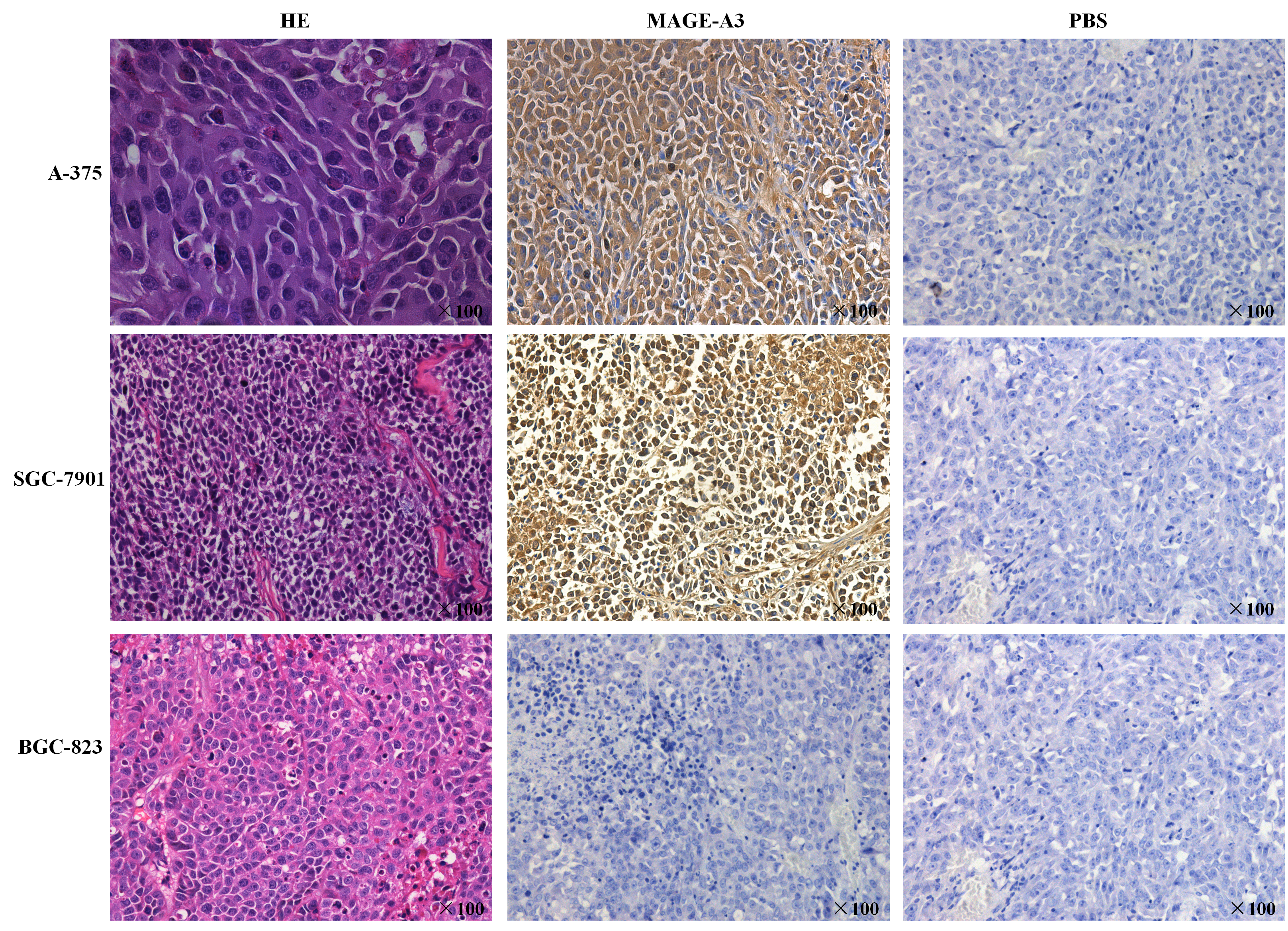


Fig.S10 Immunohistochemical detection of gastric cancer tissue specimens in tumor-bearing nude mice. There are clusters of brown-yellow precipitate particles in the tumor cytoplasm in the A-375 and SGC-7901 tumor tissue, while a negative in BGC-823 tumor tissues and PBS group.

Supplement Table 1

Correlation analysis between MAGEA3 and relate genes and markers of immune cells in TIMER

| Description | Gene marker | None | | Purity | |
| --- | --- | --- | --- | --- | --- |
|  |  | cor | p | cor | p |
| T cell | CD3D | -0.2298 | **2.23E-06^*^** | -0.2170 | **2.04E-05^*^** |
|  | CD3E | -0.2333 | **1.55E-06^*^** | -0.2165 | **2.13E-05^*^** |
|  | CD2 | -0.2083 | **1.88E-05^*^** | -0.1892 | **2.12E-04^*^** |
| B cell | CD19 | -0.1150 | **1.91E-02^*^** | -0.1010 | **4.95E-02^*^** |
|  | CD79A | -0.1338 | **6.33E-03^*^** | -0.1192 | **2.03E-02^*^** |
| Neutrophils | CEACAM8 | 0.1219 | **1.29E-02^*^** | 0.1400 | **6.33E-03^*^** |
|  | ITGAM | -0.1098 | **2.53E-02^*^** | -0.0768 | 1.36E-01 |
|  | CCR7 | -0.1742 | **3.63E-04^*^** | -0.1460 | **4.40E-03^*^** |
| NK cell | KIR2DL1 | 0.0155 | 7.53E-01 | 0.0041 | 9.37E-01 |
|  | KIR2DL3 | -0.0023 | 9.63E-01 | 0.0012 | 9.81E-01 |
|  | KIR2DL4 | -0.0646 | 1.89E-01 | -0.0664 | 1.97E-01 |
|  | KIR3DL1 | -0.1007 | **4.03E-02^*^** | -0.1256 | **1.44E-02^*^** |
|  | KIR3DL2 | -0.0826 | 9.27E-02 | -0.0892 | 8.29E-02 |
|  | KIR3DL3 | -0.0320 | 5.16E-01 | -0.0469 | 3.62E-01 |
|  | KIR2DS4 | -0.0932 | 5.79E-02 | -0.1129 | 2.80E-02**^*^** |
| DC | HLA-DPB1 | -0.2231 | **4.45E-06^*^** | -0.1921 | **1.68E-04^*^** |
|  | HLA-DQB1 | -0.1804 | **2.20E-04^*^** | -0.1467 | **4.20E-03^*^** |
|  | HLA-DRA | -0.2218 | **5.08E-06^*^** | -0.1902 | **1.95E-04^*^** |
|  | HLA-DPA1 | -0.1882 | **1.15E-04^*^** | -0.1546 | **2.55E-03^*^** |
|  | CD1C | -0.1629 | **8.67E-04^*^** | -0.1216 | **1.78E-02^*^** |
|  | NRP1 | -0.0677 | 1.68E-01 | -0.0365 | 4.78E-01 |
|  | ITGAX | -0.0953 | 5.24E-02 | -0.0579 | 2.61E-01 |
| TH1 | TBX2 | 0.0003 | 9.95E-01 | 0.0222 | 6.67E-01 |
|  | STAT4 | -0.1310 | **7.54E-03^*^** | -0.1082 | **3.52E-02^*^** |
|  | STAT1 | -0.0103 | 8.35E-01 | -0.0052 | 9.20E-01 |
|  | IFNG | -0.1215 | **1.32E-02^*^** | -0.1224 | **1.71E-02^*^** |
|  | TNF | 0.0220 | 6.56E-01 | 0.0781 | 1.29E-01 |
| TH2 | GATA3 | -0.2129 | **1.22E-05^*^** | -0.1953 | **1.30E-04^*^** |
|  | STAT6 | -0.0801 | 1.03E-01 | -0.0656 | 2.03E-01 |
|  | STAT5A | -0.1861 | **1.38E-04^*^** | -0.1690 | **9.54E-04^*^** |
|  | IL13 | 0.0069 | 8.88E-01 | -0.0069 | 8.94E-01 |
| TfH | BCL6 | -0.0704 | 1.52E-01 | -0.0510 | 3.22E-01 |
|  | IL21 | -0.1322 | **7.01E-03^*^** | -0.1104 | **3.17E-02^*^** |
| TH17 | STAT3 | -0.0451 | 3.60E-01 | -0.0438 | 3.96E-01 |
|  | IL17A | 0.0757 | 1.23E-01 | 0.0690 | 1.80E-01 |
| Treg | FOXP3 | -0.0905 | **6.56E-02^*^** | -0.0681 | 1.86E-01 |
|  | CCR8 | -0.0391 | 4.27E-01 | -0.0251 | 6.27E-01 |
|  | STAT5B | -0.0378 | 4.43E-01 | -0.0264 | 6.08E-01 |
|  | TGFB1 | -0.0734 | 1.36E-01 | -0.0496 | 3.36E-01 |
| T cell exhaustion | PDCD1 | -0.1803 | **2.23E-04^*^** | -0.1701 | **8.82E-04^*^** |
|  | CTLA4 | -0.0864 | 7.88E-02 | -0.1091 | **2.62E-02^*^** |
|  | LAG3 | -0.1524 | **1.85E-03^*^** | -0.1488 | **3.68E-03^*^** |
|  | HAVCR2 | -0.1436 | **3.36E-03^*^** | -0.1123 | **2.88E-02^*^** |
|  | GZMB | -0.1263 | **1.00E-02^*^** | -0.1288 | **1.21E-02^*^** |
| Monocyte | CD86 | -0.1047 | **3.29E-02^*^** | -0.0686 | 1.83E-01 |
|  | CSF1R | -0.0988 | **4.42E-02^*^** | -0.0825 | 1.09E-01 |
| TAM | CCL2 | -0.0432 | 3.80E-01 | 0.0005 | 9.92E-01 |
|  | CD68 | -0.0197 | 6.88E-01 | 0.0315 | 5.40E-01 |
|  | IL10 | -0.0951 | 5.29E-02 | -0.0612 | 2.34E-01 |
| M1 Monocyte | NOS2 | -0.0795 | 1.06E-01 | -0.0862 | 9.38E-02 |
|  | IRF5 | -0.0081 | 8.69E-01 | 0.0277 | 5.91E-01 |
|  | PTGS2 | 0.0464 | 3.46E-01 | 0.0594 | 2.49E-01 |
| M2 Monocyte | CD163 | -0.0789 | 1.09E-01 | -0.0526 | 3.07E-01 |
|  | MS4A4A | -0.1477 | **2.56E-03^*^** | -0.1192 | **2.03E-02^*^** |
|  | VSIG4 | -0.0852 | 8.28E-02 | -0.0603 | 2.42E-01 |

Cor, R value of spearman’s correlation: *: p< 0.05

Supplement Table 2

Correlation analysis of MAGEA3 expression with immune checkpoint genes and chemokines/chemokine receptors, MHC.

| Description | Gene marker | None | | Purity | |
| --- | --- | --- | --- | --- | --- |
|  |  | cor | p | cor | p |
| Chemokine | CCL1 | -0.0536 | 2.76E-01 | -0.0463 | 3.69E-01 |
|  | CCL2 | -0.0432 | 3.80E-01 | 0.0005 | 9.92E-01 |
|  | CCL3 | -0.0287 | 5.60E-01 | -0.0045 | 9.31E-01 |
|  | CCL4 | -0.1407 | **4.08E-03^*^** | -0.1327 | **9.69E-03^*^** |
|  | CCL5 | -0.2199 | **6.13E-06^*^** | -0.2167 | **2.09E-05^*^** |
|  | CCL7 | 0.0772 | 1.17E-01 | 0.1026 | **4.58E-02^*^** |
|  | CCL8 | -0.0405 | 4.10E-01 | -0.0178 | 7.29E-01 |
|  | CCL11 | -0.0750 | 1.27E-01 | -0.0589 | 2.53E-01 |
|  | CCL13 | -0.0372 | 4.50E-01 | -0.0060 | 9.07E-01 |
|  | CCL14 | -0.1246 | **1.11E-02^*^** | -0.0864 | 9.29E-02 |
|  | CCL15 | -0.1587 | **1.18E-03^*^** | -0.1401 | **6.30E-03^*^** |
|  | CCL16 | -0.0828 | 9.20E-02 | -0.0834 | 1.05E-01 |
|  | CCL17 | -0.1438 | **3.33E-03^*^** | -0.1045 | **4.21E-02^*^** |
|  | CCL18 | -0.0356 | 4.69E-01 | -0.0071 | 8.90E-01 |
|  | CCL19 | -0.1633 | **8.39E-04^*^** | -0.1332 | **9.43E-03^*^** |
|  | CCL20 | 0.0862 | 7.93E-02 | 0.0846 | 1.00E-01 |
|  | CCL21 | -0.1367 | **5.29E-03^*^** | -0.1140 | **2.65E-02^*^** |
|  | CCL22 | -0.1309 | **7.59E-03^*^** | -0.1064 | **3.84E-02^*^** |
|  | CCL23 | -0.1400 | **4.27E-03^*^** | -0.0920 | 7.35E-02 |
|  | CCL24 | -0.0637 | 1.95E-01 | -0.0657 | 2.02E-01 |
|  | CCL25 | 0.0190 | 7.00E-01 | 0.0219 | 6.71E-01 |
|  | CCL26 | 0.0868 | 7.73E-02 | 0.1015 | **4.83E-02^*^** |
|  | CCL27 | 0.0587 | 2.33E-01 | 0.0734 | 1.54E-01 |
|  | CCL28 | 0.0268 | 5.86E-01 | 0.0402 | 4.35E-01 |
|  | CX3CL1 | -0.1302 | **7.91E-03^*^** | -0.0981 | 5.63E-02 |
|  | CXCL1 | 0.0068 | 8.91E-01 | 0.0243 | 6.37E-01 |
|  | CXCL2 | 0.0707 | 1.51E-01 | 0.0871 | 9.03E-02 |
|  | CXCL3 | -0.0112 | 8.21E-01 | -0.0049 | 9.25E-01 |
|  | CXCL5 | 0.0588 | 2.32E-01 | 0.0705 | 1.71E-01 |
|  | CXCL6 | 0.1367 | **5.28E-03^*^** | 0.1813 | **3.88E-04^*^** |
|  | CXCL9 | -0.1056 | **3.15E-02^*^** | -0.1004 | 5.08E-02 |
|  | CXCL10 | -0.0615 | 2.11E-01 | -0.0493 | 3.38E-01 |
|  | CXCL11 | -0.0979 | **4.63E-02^*^** | -0.0831 | 1.06E-01 |
|  | CXCL12 | -0.0698 | 1.56E-01 | -0.0287 | 5.77E-01 |
|  | CXCL13 | -0.1809 | **2.11E-04^*^** | -0.1707 | **8.50E-04^*^** |
|  | CXCL14 | 0.0451 | 3.60E-01 | 0.0723 | 1.60E-01 |
|  | CXCL16 | 0.0341 | 4.88E-01 | 0.0546 | 2.89E-01 |
|  | CXCL17 | -0.0163 | 7.41E-01 | -0.0026 | 9.60E-01 |
|  | XCL1 | 0.2107 | **1.50E-05^*^** | 0.2217 | **1.33E-05^*^** |
|  | XCL2 | -0.1493 | **2.30E-03^*^** | -0.1446 | **4.81E-03^*^** |
| Receptor | CCR1 | -0.0631 | 2.00E-01 | -0.0267 | 6.04E-01 |
|  | CCR2 | -0.1032 | **3.55E-02^*^** | -0.0716 | 1.64E-01 |
|  | CCR3 | 0.1196 | **1.48E-02^*^** | 0.1438 | **5.03E-03^*^** |
|  | CCR4 | -0.1402 | **4.21E-03^*^** | -0.1124 | **2.86E-02^*^** |
|  | CCR5 | -0.2087 | **1.82E-05^*^** | -0.1915 | **1.77E-04^*^** |
|  | CCR6 | -0.0792 | 1.07E-01 | -0.0495 | 3.36E-01 |
|  | CCR7 | -0.1742 | **3.63E-04^*^** | -0.1460 | **4.40E-03^*^** |
|  | CCR8 | -0.0391 | 4.27E-01 | -0.0251 | 6.27E-01 |
|  | CCR9 | -0.0801 | 1.03E-01 | -0.0698 | 1.75E-01 |
|  | CCR10 | 0.0064 | 8.96E-01 | 0.0177 | 7.31E-01 |
|  | CXCR1 | 0.0646 | 1.89E-01 | 0.1038 | **4.34E-02^*^** |
|  | CXCR2 | 0.0761 | 1.21E-01 | 0.1099 | **3.25E-02^*^** |
|  | CXCR3 | -0.2412 | **6.62E-07^*^** | -0.2274 | **7.76E-06^*^** |
|  | CXCR4 | -0.1122 | **2.23E-02^*^** | -0.0913 | 7.60E-02 |
|  | CXCR5 | -0.1974 | **5.13E-05^*^** | -0.1786 | **4.76E-04^*^** |
|  | CXCR6 | -0.1879 | **1.17E-04^*^** | -0.1736 | **6.89E-04^*^** |
|  | XCR1 | -0.1535 | **1.72E-03^*^** | -0.1399 | **6.38E-03^*^** |
|  | CX3CR1 | -0.0703 | 1.53E-01 | -0.0493 | 3.39E-01 |
| MHC | B2M | -0.2091 | **1.76E-05^*^** | -0.1759 | **5.83E-04^*^** |
|  | HLA-A | -0.0684 | 1.64E-01 | -0.0585 | 2.56E-01 |
|  | HLA-B | -0.1418 | **3.80E-03^*^** | -0.1305 | **1.10E-02^*^** |
|  | HLA-C | -0.0695 | 1.58E-01 | -0.0469 | 3.63E-01 |
|  | HLA-DMA | -0.2216 | **5.18E-06^*^** | -0.1848 | **2.99E-04^*^** |
|  | HLA-DMB | -0.1995 | **4.28E-05^*^** | -0.1612 | **1.64E-03^*^** |
|  | HLA-DOA | -0.1707 | **4.80E-04^*^** | -0.1317 | **1.03E-02^*^** |
|  | HLA-DOB | -0.1783 | **2.61E-04^*^** | -0.1545 | **2.55E-03^*^** |
|  | HLA-DPA1 | -0.1882 | **1.15E-04^*^** | -0.1546 | **2.55E-03^*^** |
|  | HLA-DPB1 | -0.2231 | **4.45E-06^*^** | -0.1921 | **1.68E-04^*^** |
|  | HLA-DQA1 | -0.1863 | **1.35E-04^*^** | -0.1491 | **3.62E-03^*^** |
|  | HLA-DQA2 | -0.1242 | **1.13E-02^*^** | -0.1122 | **2.90E-02^*^** |
|  | HLA-DQB1 | -0.1804 | **2.20E-04^*^** | -0.1467 | **4.20E-03^*^** |
|  | HLA-DRA | -0.2218 | **5.08E-06^*^** | -0.1902 | **1.95E-04^*^** |
|  | HLA-DRB1 | -0.2093 | **1.72E-05^*^** | -0.1813 | **3.88E-04^*^** |
|  | HLA-E | -0.1878 | **1.19E-04^*^** | -0.1633 | **1.43E-03^*^** |
|  | HLA-F | -0.1478 | **2.54E-03^*^** | -0.1247 | **1.51E-02^*^** |
|  | HLA-G | 0.1511 | **2.03E-03^*^** | 0.1572 | **2.14E-03^*^** |
|  | TAP1 | -0.1140 | **2.02E-02^*^** | -0.0978 | 5.71E-02 |
|  | TAP2 | -0.0895 | 6.84E-02 | -0.0658 | 2.01E-01 |
|  | TAPBP | -0.0521 | 2.90E-01 | -0.0341 | 5.08E-01 |
| Immunoinhibitor | ADORA2A | -0.0305 | 5.35E-01 | 0.0003 | 9.96E-01 |
|  | BTLA | -0.1905 | **9.39E-05^*^** | -0.1767 | **5.51E-04^*^** |
|  | CD160 | -0.0245 | 6.18E-01 | -0.0457 | 3.75E-01 |
|  | CD244 | -0.1177 | **1.65E-02^*^** | -0.1178 | **2.18E-02^*^** |
|  | CD274 | -0.1561 | **1.42E-03^*^** | -0.1487 | **3.71E-03^*^** |
|  | CD96 | -0.2079 | **1.96E-05^*^** | -0.2016 | **7.73E-05^*^** |
|  | CSF1R | -0.0988 | **4.42E-02^*^** | -0.0825 | 1.09E-01 |
|  | CTLA4 | -0.0864 | 7.88E-02 | -0.1091 | **2.62E-02^*^** |
|  | HAVCR2 | -0.1436 | **3.36E-03^*^** | -0.1123 | **2.88E-02^*^** |
|  | IDO1 | -0.1027 | **3.65E-02^*^** | -0.0886 | 8.48E-02 |
|  | IL10 | -0.0951 | 5.29E-02 | -0.0612 | 2.34E-01 |
|  | IL10RB | 0.0127 | 7.97E-01 | 0.0237 | 6.45E-01 |
|  | KDR | -0.0838 | 8.82E-02 | -0.0568 | 2.70E-01 |
|  | KIR2DL1 | 0.0155 | 7.53E-01 | 0.0041 | 9.37E-01 |
|  | KIR2DL3 | -0.0023 | 9.63E-01 | 0.0012 | 9.81E-01 |
|  | LAG3 | -0.1524 | **1.85E-03^*^** | -0.1488 | **3.68E-03^*^** |
|  | LGALS9 | -0.2861 | **2.92E-09^*^** | -0.2579 | **3.58E-07^*^** |
|  | PDCD1 | -0.1803 | **2.23E-04^*^** | -0.1701 | **8.82E-04^*^** |
|  | PDCD1LG2 | -0.1281 | **9.01E-03^*^** | -0.1020 | **4.71E-02^*^** |
|  | PVRL2 | 0.1445 | **3.17E-03^*^** | 0.1480 | **3.88E-03^*^** |
|  | TGFB1 | -0.0734 | 1.36E-01 | -0.0496 | 3.36E-01 |
|  | TGFBR1 | 0.0305 | 5.36E-01 | 0.0232 | 6.53E-01 |
|  | TIGIT | -0.1581 | **1.23E-03^*^** | -0.1416 | **5.75E-03^*^** |
|  | VTCN1 | 0.1633 | **8.38E-04^*^** | 0.1673 | **1.08E-03^*^** |
| Immunostimulator | BTNL2 | -0.0045 | 9.27E-01 | -0.0016 | 9.76E-01 |
|  | C10orf54 | -0.0874 | 7.55E-02 | -0.0623 | 2.26E-01 |
|  | CD27 | -0.1697 | **5.17E-04^*^** | -0.1534 | **2.76E-03^*^** |
|  | CD276 | 0.1169 | **1.72E-02^*^** | 0.1232 | **1.64E-02^*^** |
|  | CD28 | -0.1263 | **1.00E-02^*^** | -0.0977 | 5.74E-02 |
|  | CD40 | -0.0259 | 5.99E-01 | 0.0189 | 7.13E-01 |
|  | CD40LG | -0.2038 | **2.88E-05^*^** | -0.1794 | **4.48E-04^*^** |
|  | CD48 | -0.2299 | **2.22E-06^*^** | -0.2042 | **6.22E-05^*^** |
|  | CD70 | -0.0944 | 5.46E-02 | -0.0725 | 1.59E-01 |
|  | CD80 | -0.0565 | 2.51E-01 | -0.0344 | 5.04E-01 |
|  | CD86 | -0.1047 | **3.29E-02^*^** | -0.0686 | 1.83E-01 |
|  | ENTPD1 | -0.1159 | **1.82E-02^*^** | -0.0897 | **8.12E-02^*^** |
|  | HHLA2 | 0.1041 | **3.40E-02^*^** | 0.1300 | **1.13E-02^*^** |
|  | ICOS | -0.0982 | **4.55E-02^*^** | -0.0661 | 1.99E-01 |
|  | ICOSLG | -0.0967 | **4.90E-02^*^** | -0.0669 | 1.94E-01 |
|  | IL2RA | -0.1125 | **2.19E-02^*^** | -0.0929 | 7.09E-02 |
|  | IL6 | -0.0009 | 9.85E-01 | 0.0271 | 6.00E-01 |
|  | IL6R | 0.0105 | 8.31E-01 | 0.0266 | 6.05E-01 |
|  | KLRC1 | -0.1321 | **7.03E-03^*^** | -0.1472 | **4.07E-03^*^** |
|  | KLRK1 | -0.2400 | **7.57E-07^*^** | -0.2316 | **5.22E-06^*^** |
|  | LTA | -0.1973 | **5.18E-05^*^** | -0.1797 | **4.37E-04^*^** |
|  | MICB | -0.0227 | 6.45E-01 | -0.0153 | 7.66E-01 |
|  | NT5E | 0.0282 | 5.67E-01 | 0.0360 | 4.84E-01 |
|  | PVR | 0.2410 | **6.77E-07^*^** | 0.2244 | **1.03E-05^*^** |
|  | RAET1E | 0.0380 | 4.41E-01 | 0.0629 | 2.22E-01 |
|  | TMEM173 | -0.2140 | **1.10E-05^*^** | -0.1905 | **1.91E-04^*^** |
|  | TMIGD2 | -0.1938 | **7.10E-05^*^** | -0.1816 | **3.80E-04^*^** |
|  | TNFRSF13B | -0.1630 | **8.60E-04^*^** | -0.1546 | **2.54E-03^*^** |
|  | TNFRSF13C | -0.1197 | **1.47E-02^*^** | -0.1122 | **2.89E-02^*^** |
|  | TNFRSF14 | -0.1091 | **2.63E-02^*^** | -0.0704 | 1.71E-01 |
|  | TNFRSF17 | -0.0771 | 1.17E-01 | -0.0715 | 1.65E-01 |
|  | TNFRSF18 | 0.0372 | 4.50E-01 | 0.0450 | 3.83E-01 |
|  | TNFRSF25 | 0.1300 | **8.04E-03^*^** | 0.1403 | **6.23E-03^*^** |
|  | TNFRSF4 | -0.0760 | 1.22E-01 | -0.0483 | 3.49E-01 |
|  | TNFRSF8 | -0.0679 | 1.68E-01 | -0.0479 | 3.53E-01 |
|  | TNFRSF9 | -0.1272 | **9.46E-03^*^** | -0.1113 | **3.03E-02^*^** |
|  | TNFSF13 | -0.1379 | **4.89E-03^*^** | -0.1136 | **2.70E-02^*^** |
|  | TNFSF13B | -0.1695 | **5.24E-04^*^** | -0.1507 | **3.27E-03^*^** |
|  | TNFSF14 | -0.1686 | **5.63E-04^*^** | -0.1432 | **5.22E-03^*^** |
|  | TNFSF15 | -0.0041 | 9.34E-01 | -0.0017 | 9.74E-01 |
|  | TNFSF18 | 0.0541 | 2.72E-01 | 0.0540 | 2.95E-01 |
|  | TNFSF4 | 0.0049 | 9.21E-01 | 0.0165 | 7.48E-01 |
|  | TNFSF9 | -0.2000 | **4.07E-05^*^** | -0.2036 | **6.52E-05^*^** |
|  | ULBP1 | 0.2817 | **5.23E-09^*^** | 0.2718 | **7.62E-08^*^** |

Cor, R value of spearman’s correlation: *: p< 0.05
